# Voters reveal public library value: Evidence from US referendums

**DOI:** 10.1371/journal.pone.0351352

**Published:** 2026-10-01

**Authors:** Margo Gustina, Robert P. Berrens

**Affiliations:** 1 College of University Libraries and Learning Sciences, University of New Mexico, Albuquerque, New Mexico, United States of America; 2 Department of Economics, University of New Mexico, Albuquerque, New Mexico, United States of America; University of Amsterdam, NETHERLANDS, KINGDOM OF THE

## Abstract

Qualitative research has shown public libraries to be well-loved institutions. But, as they are increasingly under attack in the US, quantitatively understanding the drivers of public voting support can help policy makers establish allocation priorities. Using novel voter response datasets (2010–2022) at the library system-year level nationally (N = 982), and at the precinct-year level within Washington state (N = 5,380), referendum modeling approaches are used to investigate local preferences for public libraries. Further, this study probes environmental condition effects on preferences. Results indicate that with precinct-year level estimations, voter preferences for facility access overwhelm other effects. Where a precinct contains a library building, the probability of a Yes vote increases 13%. Library support decreases 4% for every additional mile traveled to a location and is sensitive to extreme heat events. Taken together, the results demonstrate significant empirical support for libraries as critical infrastructure.

## Introduction

Public spaces can have multidimensional effects across socio-ecological systems [1]. In the United States (US), urban and regional planners, and other scholars, have sounded the alarm that the amount and equitable distribution of such public assets has been declining [2,3]. Nationwide there is a decline in non-privatized public spaces that offer interior access without fee-for-use payment or membership requirements, to be used for leisure, learning, or safely getting out of the weather. Public libraries are both the most common of these local resources [4] and one of the most currently contentious [5].

In much of the US, public libraries are not centrally or federally funded. Rather, statutory frameworks in many US states require, or allow, library operating and capital budgets to be set and periodically renewed through direct ballot referendums in which residents vote Yes or No on a proposed property tax levy. This institutional arrangement, uncommon relative to other public goods and outside the US, means that referendum outcomes constitute a direct, repeated, revealed-preference signal of local demand for library service, distinct from either legislative appropriation or general taxation. We make use of this revealed preference data to pursue our valuation strategy.

While congestible, public libraries offer a space where individual visitors benefit according to their capabilities and needs, groups can have shared experiences, and the community derives existence value from the assurance that the library, as a publicly accessible space, will continue to be available regardless of whether any given resident uses it directly. Public space value, which is determined by the specific local provision, its users, and the community, is not captured in any single market [6]. The multi-level and multi-dimensional nature of outcomes related to the existence of a public space to place-making, social attachment, natural disaster harm mitigation, civic action, and institutional strength, requires a similarly supra-outcome valuation method. In the case of US public libraries specifically, valuation investigations remain extremely limited, notwithstanding a broader global increase in library valuation research since the mid-1990s (detailed below). There are, however, a relatively large number of referendums on funding every year in the US that offer a pathway to public library valuation, inclusive of benefits to both users and non-users.

The objective of this study is to use available public library referendum voting event data in the US, at multiple scales, to investigate the revealed preferences of voters. The analytical model positions libraries as a place-based public good, and so, includes measures of environmental risk and spatial characteristics. Recognizing public libraries as disaster response infrastructure is common in the practice of public librarianship [7,8]. Beyond acute storm-driven disasters like tornadoes and hurricanes, public libraries are often designated as community cooling centers. This designation has captured the research interest of ethnographers [9] and a more recent causal analysis [10]. The inclusion of the environmental risk in the form of high heat days in the present analysis adds to the emerging thread in the library valuation literature.

There exists one other national study of library referendums that descriptively uses probabilities to discuss the community and library service factors that predict voter behavior [11]. Diverging from that descriptive analysis, this econometric study is conducted at two levels and scales. It begins with an analysis of the probability a public library system will pass a referendum as a function of tax incidence (the amount a household pays in annual taxes), referendum features, library system services, partisanship, and socio-ecological factors, for all confirmed public library referendum events 2010–2021 in the US using a general linear model estimated by weighted maximum likelihood with link function.

This analysis is then repeated at the precinct-year level within Washington state, 2010–2022. Washington state government’s approach to publicly available transparent data provides the researcher with a unique opportunity to do precinct-level investigation of vote events with results. Further, Washington has an institutional landscape that allows for robust analysis: uniform full-assessment tax base on which library taxes are levied and moderately high library referendum frequency in rural, suburban, and urban districts.

At the national level, results align roughly with McNutt et al. [11], but with demonstrated strength in geographic determinants and institutional context. Scaling down to Washington state precinct-year level analysis, the importance of the hyper-local library facility becomes evident.

## Background literature

Empirical studies of value can take many forms, with most studies of library value using phenomenological methods (i.e., personal experiences), or market analogues. However, one can conceptualize a public referendum on public library funding via compulsory tax as an opportunity for voters to reveal their true preferences for *the library*. There is a growing literature using real referendum data to understand the factors influencing voter preferences for nonmarket or publicly provided goods. Some have enough observations of precinct-level behavior to estimate the mean public willingness to pay (WTP) at the ballot box for the good. Within librarianship there exists robust descriptive analyses of local vote events and voter behavior [11,12], professional trade reporting on vote trends nationally (see “library referenda” category of *Library Journal*), two national survey-based studies [12], and one national analysis of actual public library vote data [11]. All are advocacy framed. None ask, what does voter behavior tell us about public library value(s)?

### Nonmarket valuation of public spaces

Koohsari et al. [13], among others, highlight the definition of public space as constructed in dialog (intentionally or not) with broader planning trends, local culture, financial and environmental resources. Some definitions are inclusive of privately-owned but publicly accessible spaces, like shopping malls, outdoor food courts, and sidewalk patios [14,15]. While these private-public spaces confer access, they operate against equitable distribution of positive externalities.

Survey-based stated preference approaches, such as contingent valuation (CV) studies, have been one of the primary methods of eliciting individual values for changes in public space, environmental, and other nonmarket goods [16]. Although the structure of such approaches for eliciting values varies, including choice experiments, payment cards, and donation-based payment schemes, a common approach elicits value via a hypothetical public referendum for a coercive tax to pay for a change in a nonmarket good like a new park [17].

There are multiple empirical challenges when adapting estimation techniques from hypothetical referendums to actual ballot conditions. The nature and structure of these data are different. For instance, in the survey environment, the precise tax amount is given to each survey respondent (i.e., $50), and then randomly varied across respondents to reveal willingness to pay [18]. But ballot language typically gives tax in terms of rates (i.e., $0.50 per $1,000 of assessed property value), which the voter must translate for themself.

Nearly all taxation schemes are endogenous with income, creating specification concerns. Estimating the marginal utility of income is necessary to calculate WTP for the budget constrained voter. From which data should this estimation be derived: tax incidence or income? Tax incidence, or the amount of money the voter can be expected to pay, is directly determined by the wealth of the voter either because it is a property tax (like most library taxes), or because it is a consumption tax with greater consumption assumed at greater levels of wealth.

With actual library votes specifically, there are problems of variation in vote outcomes. In CV-based hypothetical referendums, WTP is calculated from the results of a logistic regression that requires the outcome vote variable to be a binary: 1 for Yes, 0 for No for each survey response. In the Washington vote data, of the 5,824 precinct-years included, 73% have a majority Yes votes. Given only proportion information available for each precinct, estimation here follows guidance from Papke and Wooldridge [19] and implemented in a study of California green space referendums [20]. This study is the first to apply WTP estimation to actual library referendums.

### Public library valuation

Public libraries are often seen as having inherent value, previously holding the honor of most trusted institution in America [21]. For this reason, valuation studies with monetary equivalencies have been seen as irrelevant to library operations, and worse, to cheapen the institution. But, between 2019 and 2020 alone, 596 libraries have permanently closed in the US, an 111% increase over previous annual averages [22]. Libraries that remain open have also faced a documented escalation in formal requests to remove or restrict books from their circulating collections, an increase of nearly 30% in 2022 alone, itself the highest annual total on record up to that point [23]. Valuation can demonstrate not only the community benefit of having a public library but also what is lost when closed.

Public library service in the US is heterogenous within some set bounds (books, computers, facility, programs), and their settings are also heterogenous. There is no federal direct funding to non-tribal public libraries, nor is there federal governance over them. The federal data collection and grant making Institution of Museum and Library Services sends funds to states who then determine and govern distribution. States where funding (taxation) policies and formal regulatory definitions of public library are set. State control has led to widely varying policies and funding contexts. Finally, at the library level there are variations within states for library funding source, service population boundaries, resource allocation, and governance structure [22]. Public libraries having more heterogenous institutional settings than service provisions create significant challenges for valuation at the national scale.

Valuation of public library service isn’t new. For example, a taxation concern about value for the taxpayer dollar crops up in the earliest American library conference proposals [24]. Benjamin Franklin’s *Junto Club* may have been the first American public library group grappling with collective provision and its funding [25]. More recently, since the mid-1990s there was an increase in valuation of public library studies in the US and globally, with national trends toward benefit-cost analysis (BCA), return on investment (ROI) analysis, and privatization of government provision. Since 1997 there have been 88 peer-reviewed published library valuation studies, where the majority were conducted outside the US, in nations with more institutionally uniform library provision. Notably, 40 of them happen in nations or contexts with homogenous institutional structures like South Korea [26,27] and Norway [28,29], both of which have standardized federal library provision rules.

These two nations (South Korea and Norway) are also home to two of the three post-2000 meta-analyses on library valuation studies. As Aabø [28] notes, meta-analysis allows the estimation of either a point or range value as a generalizable value figure, even if the included studies use a variety of techniques or instruments and were conducted in diverse settings [28]. In the US, Project Meta was launched in 2013, published one comprehensive report of the challenges of meta-analysis with currently available studies, and then was discontinued [30]. Public library research conducted by economists and librarians in the US has instead focused on demonstrating impacts in areas like educational effectiveness [31]. The Aabø 2009 meta-analysis based on CV studies that result in an ROI value figure is the most recent meta-analysis of public library valuation to be published in English [28].

### Library funding referendum context

Each state in the US has its own policies regarding the administration and public provision of library services. Ebdon et al. [32] try describing the heterogeneity of these contexts in a study of the relationship between funding source types and per capita operational spending, that lead to a nuanced but not exhaustive understanding of funding referendum behavior.

For example, New York state has the greatest number of vote events with some of the lowest per household tax incidence. In this divergent finding, the many factors that influence calculation are evident. Within this one state there are multiple jurisdictional boundary and administrative structure types, each with their own vote considerations with the state’s largest systems choosing against public votes as a funding mechanism. 93% of New York’s 756 public libraries are single outlets with independent governing boards made up of volunteer trustees. Written into the New York State Trustee Handbook is the sentence, “A public library is a public service, and secure, reliable, and consistent public funding that is voter-directed is the most appropriate way to pay for it. A public library is not a charity!” [33]. Within this context, the governors are institutionalized into establishing and seeking funding through public voting, of which there are three: school district bounded, municipality bounded, and unique legislatively determined boundary [34].

This context incentivizes ballot initiatives in two ways: trustees are acculturated to expect to go for a vote and communities are expected to see funding on their ballots, and once a funding level has passed, it cannot be reduced even by a subsequent failed levy increase vote – the funding level simply remains unchanged. The two tax mechanisms also explain, in part, the low tax incidence by allowing a public library to select the taxation boundary that captures the maximum number of households. Low tax incidence is also explained by selection bias: many of the state’s highest home values are in places without public votes on libraries (New York City and much of Long Island). Finally, the low tax incidence is in part due to the relatively low number of bond issues compared to operational funding of the state’s single outlet small town libraries. In New York there is annually legislated construction funding used by the libraries who may be less likely to win bond issues if the total burden of accessibility ramps, elevators, and children’s room renovations were shouldered locally [35].

Ohio, like New York, has libraries with a variety of administrative structures and service area boundaries whose specific arrangement is laid out in their articles of incorporation [36]. But rather than legislatively allocated statewide construction funding, Ohio has the Public Library Fund. This shared distribution fund was established under a different name in 1931 and currently receives 1.7% of the state’s general revenue fund that is solely built from gross tax receipts [37]. Having this source of base operating revenue means that Ohio’s local levies follow a pattern opposite from New York’s, with more large libraries seeking public votes and an increased portion of bond issues with operating levies.

Ohio’s general revenue fund has been shrinking and with it state library funding, leading to more and a greater diversity of libraries seeking funding through a public vote [38]. The timescale of that shift is larger than the study dataset used here, that shows a weakly declining trend in vote events. Ohio has robust support for public library trustees and makes available resources and toolkits for helping administrators bring funding issues before the public [39]. This two-tiered system for funding library operations may create confusion over who pays for local services. This is one possible explanation for lower portion of Yes voters in Ohio relative to New York [12].

Southeastern and gulf states (except Florida) share a common county-based structure but with state-by-state differences in funding, resource sharing, governance, and regulation. Louisiana allows a board and its governing authority to place library funding on a ballot [40]. There are specific governing authorities unique to Louisiana which occur at the parish (county analog) level and are (mostly) either Council Presidents or Police Jury Associations. Despite its uncommon name, Police Jury Associations function much like a county commission [41]. The parish base means that all Louisiana public library referendums were conducted at either a single parish geography or, in one case of a consolidated authority, a three-parish grouping.

Determination of funding becomes a matter for each parish authority to either allocate funds directly in the budget, or to host a public referendum of a designated property tax millage. Like in other states discussed, the mills can be bond issues or operational and both have term limits. In these data, all but the New Orleans Public Library vote have one-decade terms [42]. Here, votes are more contentious with an average 62% in favor compared to New York’s 76%. But some differences are structural, as shown in the mapped distribution of vote event characteristics in Fig A1 (Appendix A).

Of Louisiana’s 68 public library systems, nearly a quarter have a public vote during our study period, with repeat referendum asks occurring for different purposes (capital construction bond and operating separately) or because the library has multiple active funding millages that expire in different years. Only two funding referendums failed to attain majority support, and both were alternate cycle millage renewals. This may indicate that residents in a moderate per capita library funding state set clear boundaries on asks.

Washington state, like most high-frequency referendum states, primarily funds public libraries with local property taxes. Washington is a full assessment state, where 100% of market value for a home is used for tax assessment. It makes Washington property tax referendums convenient for estimation of tax incidence. The WA Office of Financial Management notes that home property values have steadily increased since the 2011 recession [43]. Even though the state has a policy landscape favorable to public referendums, the year with the most libraries with operational funding requests on the ballot was 2010. Since 2011, with rising housing values, public library revenues have increased annually, without returning to the voters for increased levy rates.

The study period (2010–2022) corresponds to a period of rapid gentrification in rural Washington [44]. The movement of high-income households into rural locales shows up in these results as increasing wealth with distance from public library facilities. Property values increase with demand from high-income buyers, driving up the absolute price tag on libraries paid for through property taxes. This means that the library is more expensive for everyone, while also increasing library funding, which might mean improving service for everyone.

Within most states ballot language is standardized but tends to include different information. For instance, in Louisiana the ballots include the total funds that will be collected over the term, the total millage, and the increase in millage if the vote is not for renewal at the same levy rate. The voter here is given complete financial information. The following is representative of parish ballots:

Shall the Parish of East Baton Rouge, State of Louisiana (the “Parish”) be authorized to renew the levy and collection of 11.1 mills ad valorem tax (the “Tax”) (an estimated $43,586,820 is reasonably expected to be collected from the levy of the Tax for an entire year), for a period of ten (10) years, beginning with the tax collection for the year 2016, and annually thereafter, to and including the year 2025, and shall the proceeds of the Tax (after paying reasonable and necessary expenses of collecting and administering the Tax) be used entirely and exclusively for the purpose of maintaining and operating the public library system of the Parish and its branches, and improving services, acquiring land and constructing new library facilities, title to which is and shall be in the public, and shall the avails or proceeds of the Tax be administered by the East Baton Rouge Parish Library Board of Control, in the manner required by law?

[42]

When conceptualizing *actual* referendums as compared to valuation surveys that use hypothetical referendum language, the information difference problems become obvious. Where surveys can be designed to elicit responses from each subpopulation and sampled to approximate the socio-demographic mix of a region, there is no controlling who shows up to vote on a referendum. The characteristics of specific actual voters are unknown. There are two national surveys that can inform this study, though: (i) Current Population Survey with vote supplement (CPS); and (ii) Voter Perceptions of Public Libraries from OCLC [12].

The CPS voter supplement is conducted every other year [45]. These data are not finely geocoded in less dense areas of the US, so it cannot be used to consistently match state voting participation behavior (and wealth and education characteristics) with referendum results in the library vote dataset. Still, it evidences a systematic difference in wealth distribution between voters (higher density of high-wage earners), and non-voters (dominated by low-wage earners) as shown in Fig C1 in Appendix C.

Systematic differences between voters and non-voters is consistent with near universal majority-Yes voting results for libraries, within the national dataset, as well responses from the OCLC support for libraries perception survey [12]. In that survey study, library funding supporters tended to be middle-aged property owners. Library users noted as having the most barriers to voting Yes, were categorized as “financially strapped” [12].

Residents who select into voting create a sample that could inflate valuation estimates if each ballot cast was tied to the voter’s marginal utility of income. Were that the case, because voters tend to be wealthier, the marginal utility of income would be estimated lower than the median population utility. Because higher wealth increases the likelihood of library support, the probability of a Yes vote would be estimated higher than the median support. In this analysis, valuation estimates are taken using precinct level aggregation for income that mitigates the bias presented by in-precinct variations between voters and non-voters.

State level heterogeneity informs the underlying mechanisms for our research hypotheses. New York’s low tax incidence and high approval rate illustrate the tax price mechanism tested in H_1_; and Louisiana’s more contentious vote results and repeat-referendum pattern illustrate the need for both institutional and fixed-effect controls included in our estimation model; finally, Washington’s full-assessment property tax structure and gentrification-driven wealth patters motivate inclusion of more (potentially) exogenous spatial and education hypotheses H_2_ and H_4_.:

*H*_*1*_ *: Increasing taxes = Decreasing probability of a Yes*

Tax Incidence: The tax calculated to be borne by the voter can be conceptualized as the price for the library good, which assumes a downward sloping demand curve.

*H*_*2*_ *: Increasing distance to a library = Decreasing probability of a Yes*

Spatial: Qualitative studies of library perception of value demonstrate that people experience the library as a specific place, not a system of places [46]. Valuation studies using the travel cost method are based in the assumption that travel is costly [16].

*H*_*3*_ *: Increasing Republican vote share = Decreasing probability of a Yes*

Partisanship: Public goods and taxation vote studies indicate that as the share of Republican party voters increases, the probability of a Yes decreases [47]. Both extant library vote studies show no effect of Republican voters [11,12]. If the effect here is negative, it provides evidence that precinct-level voting data provides the necessary precision to describe voter characteristic effects lost in library system level aggregations.

*H*_*4 (a and b_)*_ *: Increasing wealth and education = Increasing probability of a Yes*

Household: Library voter perception studies [11,12] and public environmental good studies [20,48] indicate that wealth and educational attainment predict positive voter support, with age and household composition having null or ambiguous relationships.

Hazards like air quality or extreme heat are novel for their inclusion in library voting studies, and ambiguous in expected effects. Disaster response literature sometimes includes public library buildings as critical infrastructure [7], while studies that look at perceptions of risk and investment in mitigation strategies find contraction in household willingness to invest in public rather than household benefits [49]. Complicating prediction is the overlapping but distinct populations who tend to vote on local issues, populations who tend to use the public library, and populations described as most vulnerable to the hazards like air pollutants and extreme heat.

*H*_*5a*_ *: Increasing environmental hazards = Increasing probability of a Yes if voters integrate risk mitigation into their library valuation*
*H*_*5b*_ *: Increasing environmental hazards = Decreasing probability of a Yes if voters don’t conceptualize the library as a risk mitigation benefit*

Environment: Voters tend to be older, with some portion being vulnerable to extremes in pollution and heat. Low-income individuals tend to vote less often but have a higher level of vulnerability. Houseless individuals are restricted from access to voting by numerous barriers, but many use library services and are highly vulnerable [50]. Thus, the direction and magnitude of negative environmental outcomes effect on the probability of a Yes is ambiguous.

## Methods

### Data

To identify vote events nationally, multiple data sources were consulted and combined to create a library system – month – year data set: state library agencies and their collected reports from public libraries, the political consulting firm EveryLibrary, the publication Library Journal who conducts an annual survey of vote events, an open call survey conducted by the author, and Ballotpedia. These were then combined with library service statistics taken from the Public Library Survey, managed by the Institute of Museum and Library Services (IMLS).

Library systems serve heterogeneous geographies (Fig A2 in Appendix A). Boundary approximations are stated in each system’s Public Library Survey response. Where a system served multiple counties, the unweighted averages were used. This national dataset has 1,679 vote events, for 802 libraries, including 1,136 vote events with enough information that tax incidence could be calculated for 479 unique libraries.

For the analysis within Washington state, the primary list of vote events was given by the state library and supplemented through Ballotpedia. Precinct level results were taken from county Board of Election websites. Pairing precincts to the corresponding Census geography was done in ArcGIS with Washington state provided boundary files. Block group level estimates for the American Community Survey were available from 2013 through 2022 and provide the most precise population characteristic matching with precincts. 2010–2012, tracts were used. In both cases, precincts are not drawn to match Census boundaries, so the block or tract found through GIS analysis to have the greatest overlap is the population statistics matched to that precinct.

Identifying the nearness of a public library building to a voting precinct was completed using spatial near analysis that calculated the miles from the precinct edge to the closest building. The data used to identify the proximity of the library to the precinct excluded bookmobiles and non-public administrative buildings. Library building location data is included in the Public Library Survey. The data examined has 5,824 precinct-year observations for 39 operational funding vote events for 19 library systems in Washington. In this data, tax incidence is only missing for specific precincts without Census-based median house value estimates; so, no additional libraries are dropped from analysis due to incalculable tax amount.

Partisanship (i.e., political party affiliation) features prominently in referendum studies and was sourced differently for each dataset. The national dataset incorporates the MIT Election Science and Data Lab Presidential Election County Level files [51]. Within Washington, only precinct level results from the 2012 general election forward are available, so the partisanship variable was constructed using the same years in the national dataset. The primary partisanship variable is an unweighted average of percent Republican candidate votes cast in 2012, 2016, and 2020. Precinct level results were taken from the WA secretary of state [52].

Environmental data were taken from multiple sources. Data was added at the tract level for Washington state. For the national analysis, data was added for single outlet library systems serving any geography other than county; county level data was used for all others. Air quality can impact health and quality of life differently depending on its cause and intensity [53]. The air quality measure of concentration of airborne particulate matter measuring 2.5 micrometers (PM 2.5) was chosen. PM 2.5 levels categorized as “Unhealthy for Sensitive Groups” by the US Environmental Protection Agency is the measure identified by the Washington Air Monitoring Coordinator as reaching concentrations high enough, close enough to land surface, to be wildfire driven [54]. This dangerous pollutant has other causes as well, which impact outdoor air quality. The variable used is the annual count of days where PM 2.5 concentration is within the selected risk category [55].

The US Centers for Disease Control with the National Environmental Public Health Tracking Network make available historic data from North American Land Data Assimilation System (NLDAS-2) on both absolute surface temperatures and relative (heat index) temperatures. Historical annual counts of extreme heat days above specific thresholds given in 5-degree increments from 90 to 105, is downloaded for all Census tracts and counties for years 2010–2022 [56]. Heat Index rather than absolute figures at the 90- and 100-degree thresholds are used because voters are likely driven by their experience (captured in the feels-like value of the heat index) more than absolute values. These data are not available in Alaska.

A key variable is the constructed tax incidence (*TAX_INCID*). Tax incidence is the amount a household can expect to pay in annual taxes if the referendum passes. It requires assumptions about voter knowledge and behavior. Three tax incidence variables are calculated: (i) one that finds greatest consistency between states for total tax burden (*TAXINCID_TOTAL*); (ii), another that aligns with voter behavior and contingent valuation (CV) literatures [57], which calculates according to the ballot information (sometimes new tax amount, sometimes total tax amount) (*TAXINCID_NEW* or *TAXINCID_TOTAL*); and (iii) a mixed variable combining approaches (*TAXINCID_MIXED*). Further details on variable construction are provided in Appendix E, joined with a lengthy discussion of the chosen estimation strategies employed.

Bond votes are dropped from the Washington per precinct analysis dataset and retained with a fixed binary indicator in the national data. The requirements of accurate term data and clarity on tax incidence with bonds make them fundamentally different value referendums. Table B.1 gives a comprehensive listing of sources used to construct the national and Washington datasets.

### Empirical strategy

Analysis proceeds with how socio-ecological characteristics, risks, and outcomes contribute to the probability that a library tax increase wins at the ballot box using a log-odds model. Empirical research into the utility of passing a public good referendum flows from a framework established by Deacon and Shapiro [58] and others [59,60]. In that work, the dependent variable is theoretically framed as a discrete moment of choice: Yes or No to an expense. While based in that same choice moment, the structure of our data is the proportion of positive discrete decisions within an aggregated space-time boundary. Recent work on implementing estimation with fractional or proportion data consistent with the data generating process (discrete choices) has shown that intentionally selecting a distribution and applying a logit link function gives the theoretically correct and least biased results [61].

In this model, the dependent variable is the portion of Yes votes within the voting boundary and event time (full event in national data and precinct in Washington data), bounded by 0 and 1.

Independent variables used in this log-odds structure are:

$TaxIncidence_{it}$: property tax cost associated with voting Yes calculated for the geography where and when the vote occurred.
$Household_{it}$: vector of household member characteristics aggregated as proportions within the geography where and when the vote occurred (e.g., percent residents with Bachelor’s degree or more in educational attainment, BAPLUSPE).
$Environment_{it}$: vector of environmental risks and burdens as either counts or indexes associated with voting place and time.
$Partisanship_{i}$: Republican vote share for a geography averaged over presidential election events in 2012, 2016, and 2020.
$Spatial_{i}$: vector of spatial characteristics related to voting precinct and its nearest library building. This is only specified in the Washington analysis.

Time invariant variables for the institutional arrangement and context of the library system being voted on are included in the model as fixed effects.

There are a few library systems in Washington that cover enough precincts where they have enough variation themselves so that household willingness to pay (WTP) values could be estimated when the precinct results were pooled over the study years. The structure of real precinct level data can be imagined as survey results that have been collapsed by income-tax incidence level, representing a few or many hundreds of responses to a consequential payment question. For additional estimation details see Appendix E.

### Summary statistics and trends

The most prevalent story in both datasets (national and the Washington state) is that voter behavior is driven by state institutional context, environmental conditions, and geographic characteristics like level of rurality. State level policies drive the frequency of vote events, the tax mechanism, and type and level of ballot information. This heterogeneity between states is also regionally clustered in ways that may also be correlated with historic partisan boundaries. State policies that influence the frequency and content of public votes may also be regionally clustered in similar ways to administrative structures (Fig A3 in Appendix A). Table C.1 in the appendix gives state level statistics for vote events and demonstrates the challenges in measuring tax incidence with termed ballots, as is common in bond referendums. The heterogeneity of institutional arrangements necessitates careful consideration of time-invariant fixed variables. The geographic evidence is that in addition to fine grain measurement boundaries, like precincts, being valuable, limiting the scope of the geography captured to a single state may give more credible in-state results.

Political partisanship is a consideration in any study of voter behavior. While partisanship, polarization, and ideology have been shown to influence public funding and policy referendums nationally [47] and in Washington [48], the two studies of voter behavior and public library perception both indicate a null effect of Republican vote share. While the consistency of majority support in the national data supports this, there may be data precision challenges at work. Unweighted correlation scatterplots between portion of Yes voters (*p*) on library referendums against average portion of voters who voted for the Republican presidential candidate in years 2012, 2016, and 2020 (pooled) show no discernable correlative trend in the national county level presidential data as seen in Fig 1 below. Washington state shows bunching around a slight downward sloping curve. Possibly with increased geographic precision in data, more mild correlations become observable. This might also be unique to Washington state. Yet, most of precincts are voting Yes at above 40% levels, even with large numbers of voters supporting the Republican candidate.

**Fig 1:**
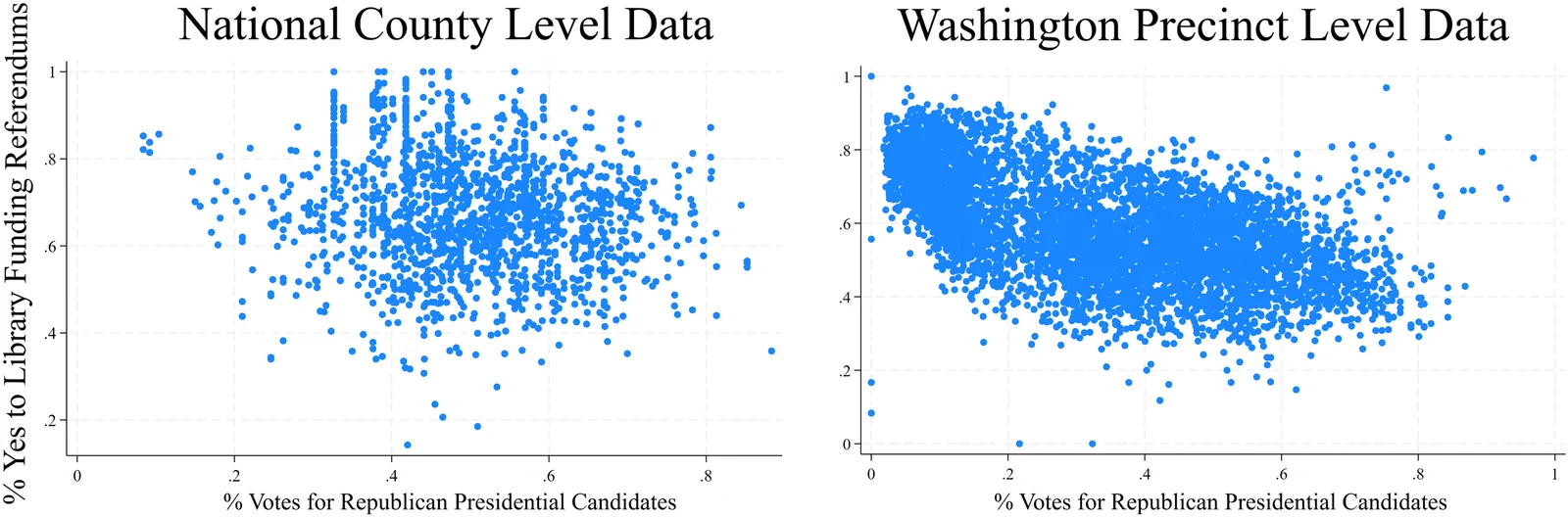
Strength of correlation between % Republican votes and Yes votes by dataset. Source. (Left) MIT Election Data and Science Lab, 2018 [51]; EveryLibrary, Library Journal, State Library Agencies of California, Michigan, New York, and Ohio, Ballotpedia, and library workers in California, Illinois, Iowa, Maryland, Nebraska, New York, South Carolina, Texas, Utah, and Wyoming. (Right) Washington Secretary of State [52] and State Library. Note. Vertical axis is % of affirmative votes for libraries and horizontal axis is portion of votes in presidential elections going to the Republican candidate. It is not evident whether the correlation pattern in the Washington data is driven by data boundary precision or by the state itself. Both represent only presidential elections pooled over 2012, 2016, and 2020.

Other national and regional exogenous events are correlated with local voter behavior. Consistent with OCLC’s 2018 national survey of voter perceptions wherein “fiscally strapped” voters were more likely to vote against increases in library funding [12], 2010 in Fig 2 below, shows the lowest voter approval rates in the national data, at both mean and outlier levels. The Great Recession of 2008 had an impact on libraries. Fiscal years 2010 and 2011 show the highest usage of libraries across use types and locations but garner the least voter support. Service and amenity use simply cannot override, entirely, the budgetary constraints.

**Fig 2:**
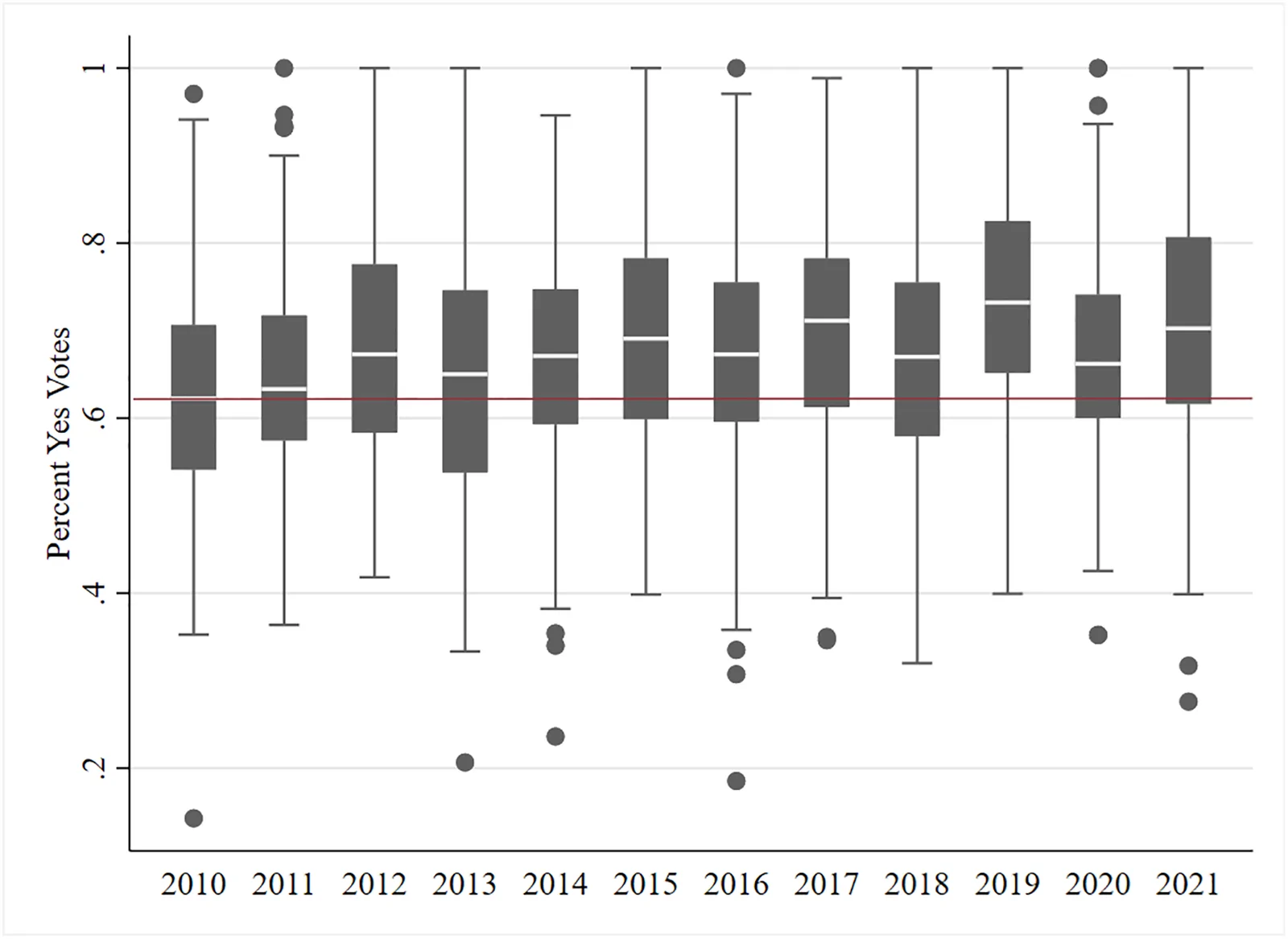
Nationwide Voter Support for Library Funding by Year of Referendum. *Sources. National vote analysis dataset*. *Notes*. Red reference line is lowest mean point of voter support, occurring in 2010, which is still above 60%. 2010 also has the lowest voter support result of all years. 2010–2011, though they saw the highest volume of public library usage, deep and widespread economic uncertainty has a strong impact on voting.

While economic uncertainty played a role in voter behavior during the Great Recession of 2008, environmental hazards, and exposure to catastrophic damages may play a stronger role over time, which could strengthen the correlation between voter support and facility closeness. Conversely, increased hazards and risk exposure could drive down support for publicly funded programs to such a level that usage and accessibility can’t ameliorate status quo voter assessment of worth. Within the Washington dataset, there is a relationship between the proportion of voters in support for the library and the nearness of a public library building to the voting precinct.

The average precinct proportion of Yes votes is much closer to 50/50 split than the overwhelming support seen at the vote event level in the national dataset. There is no clear relationship in the raw data between number of voters casting ballots in the precinct and the portion of Yes votes. The Washington dataset is limited to votes on operational funding where the tax incidence can be established, and all votes use the same taxing mechanism. These differences between datasets are evidenced in the tax incidence and odds ratio spreads. Tax incidences are between $1 and $1,630 in the national data and the much narrower $18 and $201 in the Washington data. If tax incidence was the only driver of voter behavior, we might expect a higher mean proportion of Yes votes in Washington. Other contextual drivers beyond the marginal utility of income and absolute costs are present.

## Results

Estimations are based in general linear regression modeling for distinct vote events on new or renewed property taxes to pay for public library operations. Maximum likelihood estimation is applied to the dependent variable (proportion of Yes votes), with a binomial distribution, and regressed on *logit-link-transformed* vectors of covariates describing aggregate characteristics where the vote took place. A key variable of interest is tax incidence, a calculation estimating the annual household cost of voting Yes to the average voter within the geography. Model results behave as expected if this cost or price variable shows the expected relationship: higher price should equal declining support (i.e., expected sign is negative).

The specific variables included in the regression results were chosen through an iterative process controlling for collinearity, while maintaining at least one variable from each known (and of test interest) category of voter behavior drivers: price, household, environment, partisanship, and spatial characteristics. Results proceed with estimation and interpretation of effects in national vote data and then in the Washington case study. Because these latter data are more precise, the effect of distance from voting precinct to library can be estimated. With preferred model coefficients in hand, this section concludes with a precinct level calculation of willingness to pay (WTP) with decomposition for values specific to nearest library, for two public library systems. Full regression results including fixed effect factor variables, as well as model consistency tests between possible estimation approaches (OLS and censored Tobit MLE with the log transformed dependent variable, compared to the main maximum likelihood estimated with a logit link function and Bernoulli distribution) are in Appendix F.

### What drives votes nationally

Table 1 below displays the results of regression analysis using a general linear model maximum likelihood estimation with logistic link function. The estimation approach is identical across models displayed, with iterative focus on variable groupings to explore relationships. For data sources by variable group, see Appendix B. All models are specified with fixed effects for the library system’s institutional arrangement and context: *SERVICE_GEO* refers specifically to the geography of the system’s service area where 38% of the votes included in the estimation are for city or county systems, 28% are independent taxing districts, and 20% are independent non-profits with taxation privileges. *LOCALE* is also geographic and describes the density of the library system’s service area where increasing category values means decreasing population density and increasing distance to a population center. *SINGMULT* is a binary variable indicating whether the library system is a stand-alone library with independent local governance (75%), or a multi-outlet system (25%). *NUMSTATE* is the state identifier fixed effect, which in large part determines whether and how often a library seeks a public funding referendum, which in turn drives the proportions of institutional arrangements in the estimated data. *YEAR* and *GENERAL* refer to calendar year the vote occurred in and whether the vote event happened with a general election (November), or a different month. All models are weighted by the total number of votes cast on the library funding issue (*WEIGHT* = *TOTALVOTES*). Maximum likelihood estimates with robust standard errors are provided but adjusted for the library system (*LIBKEY*) clustered effect in models 3–6. Not enough libraries in the data repeat over successive years to make a panel approach appropriate, but clustering allows the model to adjust the statistical significance for the in-group effects where libraries repeatedly go to voters.

**Table 1:** Probability Analysis of National Library Vote Data, 2010 through 2021.

	Model 1 Context	Model 2 Wealth	Model 3 Partisan	Model 4 House	Model 5 Environ.
Observations	987	987	984	984	982
Clusters			499	499	498
AIC	7710	7702	7557	7550	7428
Condition Index		6	12	34	32
TAXINCID_MIXED	−0.001**	−0.001***	−0.002***	−0.002***	−0.002**
	(0.0003)	(0.0003)	(0.0004)	(0.001)	(0.000)
MHHI_SCALED		−0.002			
		(0.0012)			
FUNDCAPITA		0.006***	0.005***	0.004**	0.004**
		(0.001)	(0.001)	(0.001)	(0.001)
AVGREP			−0.000	−0.000	−0.000
			(0.0000)	(0.000)	(0.000)
BAPLUSPE				0.003*	0.003*
				(0.002)	(0.002)
STABLEPE				−0.006**	−0.005*
				(0.002)	(0.002)
ELDHHPE				0.004	
				(0.003)	
INDEX100					0.017***
					(0.004)
SERVICE_GEO	X	X	X	X	X
LOCALE	X	X	X	X	X
SINGMULT	X	X	X	X	X
NUMSTATE	X	X	X	X	X
YEAR	X	X	X	X	X
GENERAL	X	X	X	X	X
WEIGHT	X	X	X	X	X
CLUSTERED ERRORS			X	X	X
_CONS	−0.033	−0.391	−0.0454	0.113	0.966***
	(0.299)	(0.327)	(0.322)	(0.336)	(0.281)

*Sources*. National Referendum Data 2010–2021.

*Notes*. Time-invariant variables factored out in all models are indicated with X. Robust standard errors are given in parentheses beneath coefficients. Stars indicate p values: * = p ≤ 0.05, ** = p ≤ 0.01, *** = p ≤ 0.001. Model 5 doesn’t include PM2.5 air pollution because of data availability.

As outlined in the hypotheses, tax incidence (*TAXINCID_MIXED*) is expected to be negatively driving the budget constrained voter’s decision-making at the ballot box. In all covariate-including models, for every $1 increase in tax share there is a 0.1% decrease in the likelihood of a Yes. Across the specifications the magnitude is small, but the expectation holds, except in Model 2: Wealth. This model includes household income in thousands (*MHHI_SCALED*) and the level of local funding per service population capita (*FUNDCAPITA*). Both variables have a deterministic relationship with the tax incidence. Library taxes are levied using a property tax scheme for all events included. The aggregate mean property value is strongly related to the median household income in that geography. Local funding, as reported in the Public Library Survey, operates as a lagged total tax value. Public libraries are nearly exclusively funded locally, and where tax referendums are common, local funding is almost exclusively from the most recently passed referendum. *FUNDCAPITA,* then, is the current tax burden voters bear, while simultaneously indicating a history of positive funding referendum results. When included in the model, the effect swamps the income effect, is positive (indicating culture of library support is more important than the cost of support) and with a larger magnitude than single vote event tax cost in any specification.

Partisanship (*AVGREP*) is measured as the proportion of the library system’s county that voted for the Republican presidential candidate pooled over the sample period (2012, 2016, and 2020 elections). Consistent with the only other study of library referendums, there is no statistically significant effect of partisan voting behavior on library support [11]. The present estimation only includes funding issues that use a property tax mechanism, have estimable tax incidence (excluding anomalously high burdens), and expands the sampling from 2014 through 2018–2010–2021.

Household variables describe the geography’s proportion of households that include specific characteristics, like adults with bachelor’s degrees (*BAPLUSPE*), lived at the same address 12 months ago (*STABLEPE*), and individuals over age 65 (*ELDHHPE*). These are included based on their inclusion in other studies. In the 2018 library perception study [12], higher educational attainment is correlated with higher likelihood of supporting publicly funded projects and general taxation friendliness, and older adults were more likely to use and support libraries. But this analysis is done with real referendum data at imprecise geographies. Both educational attainment and older age are correlated with going to vote that might make the relationship with voter behavior after overcoming the going to vote threshold, unvarying across vote events. *STABLEPE*, though, has statistical significance in Model 4, indicating that for every additional percentage point increase in local resident stability, the likelihood of a Yes decreases by 0.6%. Here, housing stability is assumed to proxy for home ownership. As libraries are funded by property taxes, property owners may consider tax costs more directly.

For data coverage and correlation reasons, only one kind of environmental hazard is included in estimations—for the national analysis—number of days when the heat index rose above 100°F (*INDEX100*), added in Model 5. Once included, it swamps the other aggregated household proportion effects, which were already modest or insignificant. For every day where the heat index reaches over 100°, the likelihood of Yes increases by 1.7%. This result is interpreted, as hypothesized, to indicate that where these extreme heat events are happening, voters incorporate library-as-cooling-center or other mitigating infrastructure, in their WTP valuation.

Model 5 is preferred. It uses the maximum number of vote event observations while incorporating data from each category hypothesized to influence voter behavior with a lower correlation measure than Model 4. This model specification will be followed, as closely as possible, in the Washington case study. Finally, note that the estimated coefficient on tax incidence is consistent, within overlapping confidence interval bounds, across all specifications.

The results of Model 5 indicate that given a set of 498 public libraries, some of whom make repeated requests for funding at varied intervals, the amount of the request matters to the probability of local Yes votes, along with the partisanship of the county and the frequency of extreme heat days. These relationships are apparent even when holding fixed the context of the vote (rurality, state, year) and library system structure (service geography, single or multi-outlet system). Results also provide a window into how public libraries, as built environment, are entwined with environmental conditions.

### Washington case study

Following the same iterative data analysis process used with the national dataset, Table 2 displays the results of regression analysis using a general linear model maximum likelihood estimation with logistic link function. All models are population weighted by total votes cast in the precinct (*TOTALVOTES*), with fixed effects and clustered standard errors applied differently in each model. Data sources by variable group are in the table in Appendix B.

**Table 2:** Probability Analysis of Washington Library Vote Data, 2010 through 2022.

	Model 1 Spatial	Model 2 Wealth	Model 3 Partisan	Model 4 House	Model 5 Environ.	Model 6 Pref.
Observations	5,703	5,672	5,511	5,469	5,380	5,380
Clusters			23	23		18
AIC	206	206	206	203	201	202
Condition Index	4	12	5	64	30	30
TAX_INCID	−0.001^***^	−0.002^***^	−0.001^*^	−0.001^**^	−0.001^***^	−0.003^***^
	(0.000)	(0.000)	(0.001)	(0.000)	(0.000)	(0.001)
LIBINPRECINCT	0.119^**^	0.149^***^	0.131^***^	0.141^***^	0.129^***^	0.131^***^
	(0.035)	(0.036)	(0.035)	(0.031)	(0.026)	(0.028)
DIST_MILES	−0.077^***^	−0.074^***^	−0.043^***^	−0.040^***^	−0.043^***^	−0.040^***^
	(0.007)	(0.007)	(0.010)	(0.008)	(0.005)	(0.008)
VOTENEAR_ MISMATCH	−0.163^***^	−0.167^***^	−0.108^***^	−0.113^**^	−0.119^***^	−0.111
	(0.028)	(0.028)	(0.065)	(0.040)	(0.024)	(0.064)
MHHI_SCALED		0.029^***^				
		(0.003)				
LOCFUND PERCAP		0.005^***^				
		(0.001)				
AVG_ REPUBLICAN			−0.016^***^	−0.014^***^	−0.014^***^	−0.014^***^
			(0.004)	(0.002)	(0.001)	(0.002)
BAPLUSPE				0.008^***^	0.008^***^	0.011^***^
				(0.002)	(0.001)	(0.001)
OWNEROCC_ PERC				−0.003^***^	−0.004^***^	−0.004^***^
				(0.000)	(0.000)	(0.001)
SMOCAPI_ PERC				−0.010^***^	−0.009^***^	−0.013^***^
				(0.003)	(0.002)	(0.003)
GRAPI_PERC				−0.000		
				(0.001)		
MALE_PERC				−0.000		
				(0.001)		
FEM85_PERC				0.008		
				(0.007)		
FORBORNPE				−0.008^**^	−0.007^***^	−0.007^*^
				(0.002)	(0.001)	(0.003)
POVERTY_PERC				0.001		
				(0.001)		
UNEMPLOYED_ PERC				−0.002	−0.004^**^	−0.014^*^
				(0.002)	(0.002)	(0.001)
INDEX90					0.002	0.022^*^
					(0.006)	(0.011)
UNHEALTHY PM25					−0.020^***^	−0.049
					(0.006)	(0.032)
_CONS	0.996^***^	0.329^***^	1.193^***^	1.200^***^	1.230^***^	1.230^***^
	(0.016)	(0.018)	(0.075)	(0.174)	(0.063)	(0.063)
NEAR_LOCALE	X	X	X	X	X	X
GENERAL	X	X	X	X	X	X
YEAR				X	X	
WEIGHT	X	X	X	X	X	X
CLUSTERED ERRORS			X	X		X

*Sources*. Washington Referendum Data 2010–2022.

*Notes*. Time invariant variables factored out are indicated with X. Stars indicate p values: * = p ≤ 0.05, ** = p ≤ 0.01, *** = p ≤ 0.001.

Precinct level data allows an examination not only the administrative context of the vote, but the spatial relationship of voters to the library being voted on. Model 1 estimates spatial relationship variables specifically, without clustering standard errors for the library system (*LIBKEY*). This model estimates the tax incidence (*TAX_INCID*) effect consistent with the national data, the given hypothesis, and with economic theory: for every dollar increase in estimated household tax burden, the probability of a Yes decreases by 0.1%. Spatial characteristics dominate the drivers of voting characteristics in this model, over the cost of public libraries.

Whether there is a library outlet within the precinct (*LIBINPRECINCT*), increases the likelihood of a Yes by 12%, while every mile traveled to a library outlet from the precinct edge (*DIST_MILES*) decreases the likelihood of a Yes by 8%. Only 6% of precincts included are closest to a library that is not in the overall library system with the funding referendum (*VOTENEAR_MISMATCH*). Where this occurs, voters may cast protest votes about having to fund a library system that is not *their* library, resulting in a 16% decline.

This, and all subsequent models, has two fixed effects: one for whether the vote coincided with a general election (*GENERAL*) and the level of rurality of the library outlet closest to the precinct (*NEAR_LOCALE*). The level of urban/rural captured in the National data was for the location of the administrative unit. Here, the data allows for more precise categorization of population density and commute times (by proximity).

Model 2 explores wealth measures. Tax incidence is a calculated following equation (1) as the property levy multiplied by per 1,000 property value. Household income (*MHHI_SCALED*) and house value are highly correlated, so including both it and our primary independent variable (*TAX_INCID*) creates problems of collinearity. Also included here, but not in future models because of correlation issues, is the level of tax funding per capita for the library system (*LOCFUNDPERCAP*). This can be read as a lagged indicator of voter support. Both results are positive, which is consistent with the public funding referendum literature and economic theory on the marginal utility of income. Wealthy people tend to be more supportive of library funding measures [12], and as people have more money, each additional dollar is a little less valuable. This marginal decline in the value of money as people accumulate wealth means that a tax incidence of $100 is more expensive for a household making $40,000 than it is for a house making $200,000, thereby increasing the likelihood of voting in support of public funding. Consistent with the hypothesized result, the estimation is that for every additional $10,000 in income, the voter is 3% more likely to vote Yes.

The maximum likelihood estimation reports robust standard errors, and in Model 3 these are clustered at the referendum library system level (*LIBKEY*). Although there are thousands of precincts for which we have data, there are few library systems in Washington, which can lower statistical significance. The estimated coefficient on *TAX_INCID* has a consistent sign and magnitude with other models and the lowest significance.

The focus in Model 3 is the effect of partisanship. Spatial variables retained are estimated with consistent signs, significance, and all but distance to a library (*DIST_MILES*) stay the same in magnitude. The decline in coefficient from 8% to 4% in negative effect might indicate that partisanship is correlated in Washington with rurality and distance to amenities. Partisanship is measured as the proportion of voters in a precinct who voted for the Republican presidential candidate, averaged over the 2012, 2016, and 2020 elections (*AVG_REPUBLICAN*). As hypothesized, increasing the share of voters who select Republican candidates by 1 percentage point, decreases the probability of a precinct voting Yes by 2%. The result is estimated in a model that factors out whether the vote occurred concurrent with a general election (*GENERAL*). Voter turnout is higher during general elections, but might not significantly shift partisan proportions.

Partisanship’s effect of voter behavior is slightly attenuated by the inclusion of household variables from the American Community Survey, aggregated at the tract (2010–2012) or block (2013–2022) level and assigned to the precinct with the greatest geographic overlap. The hypothesis is that anything correlated with increased wealth or education will increase the likelihood of a Yes vote, such as the portion with a Bachelor’s degree or higher (*BAPLUSPE*), or in their inverse, impoverished households (*POVERTY_PERC*), unemployed workers (*UNEMPLOYED_PERC*), and immigrants (*FORBORNPE*). Poverty may be insignificant here because it is highly correlated with status as a foreign-born resident of Washington. Immigrant share of the population grew over the period and poverty outstripped the national average by 6% with nearly one-third of households living in poverty in certain counties [62]. For every one percentage point increase in immigrant households, there is a 0.8% decline in the likelihood of Yes.

Other household and community variables were added based on their relationship to voting in the literature (proportion reporting male (*MALE*), or elder female (*FEM85*)), and to the logic of property tax voting relating to how owners and renters may view the cost of voting Yes. Self-reported sex was statistically insignificant. In survey-based studies like OCLC [12], where the individual’s preferences are tied to their reporting, such distinctions are estimated to have meaning. Aggregated at the precinct overlap level, though, there is no visible difference in preferences.

Portions of property owners (*OWNEROCC_PERC*) and households with housing costs exceeding 30% of income (*SMOCAPI_PERC*) are both significant. For every 1 percentage point increase in properties occupied by owners, there is a 0.3% decrease in the likelihood of Yes. Owners may weight the costs of property tax differently than renters, but the magnitude is small. Public libraries are neighborhood and community infrastructure, which property owners may see as beneficial.

Cost burden on owners is specified concurrently with the proportion of cost burdened renter households (*GRAPI_PERC*). Here, the renter’s burden is completely insignificant while the owner’s rent burden predicts a 1% decrease in Yes probability for every 1 percentage point increase in owner occupied households that are cost burdened.

Models 5 and 6 incorporates extreme heat days (*INDEX90*) and high air pollution days (*UNHEALTHYPM25*), at the overlapped tract level. Having precinct level voter data provides a level of spatial precision for looking at relationships between voters and local characteristics in a real setting, but environmental hazards like air quality and heat are not usually so precise in their harms. These models are specified without reported gender, poverty, or renter burden data. Coefficients estimated on the variables carried over from previous models stay consistent with only minor reduction of benefit from having a library in precinct (1.4% to 1.3%).

Key differences between models 5 and 6 are in the inclusion of year fixed effects and clustering. Model 5 includes year fixed effects but doesn’t include clustered standard errors to explore effects without the reduction in significance caused by the low number of library systems (18) that have matched environmental data. Clustering is appropriate given that the library system is the issue being considered on the ballot, but years is very correlated with the environmental hazards. Due to event sample sizes, when WTP is explored at the library system level, the data are pooled over years that had votes [48].

These two changes in specification had impacted the magnitude and significance (but not sign) of both environmental hazard coefficients. Shifting from factoring out the year of vote event to clustering standard errors moves extreme heat days from an insignificant 0.002 to a 2% increase in the probability of a Yes with 95% significance. That same specification change moves high pollution days from a 2% decrease in the probability of Yes (99.9% significance) to insignificance.

As one possible explanation, consider that the way individuals experience extreme heat is more spatially dependent than the intensity of air pollution. Heat islands and dense housing without central air conditioning coincide with more urban areas, that further coincide with higher densities of public library outlets. Voters in these environments may simply have higher frequency of relief from heat, even if they themselves are not vulnerable to the morbidity and mortality risks for unsheltered and medically compromised individuals. During the study period, these events were tied tightly to specific years, so year fixed effects subsume the variation.

By contrast, ground level wildfire smoke, as well as other dangerous pollutants, which produces high concentrations of PM2.5 in Washington is more geographically diffuse; it likely impacts at least several library system service geographies. This could create a mismatch between the ballot treatment and the hazard. And the populations experiencing the extreme edges of the harm are more geographically and demographically diverse, even though the people most vulnerable to the risks are the same. Wildfire in Washington is a regular occurrence, but impacted regions differ by year. For voters who experience uncertainty and increased costs, buying or upgrading household air filtration may constrict support for public referendums. In the specification with year fixed effects but no error clustering, for each additional smoke day, voters are 2% less likely to support a referendum.

In keeping with broader research on support for public programs and partisanship, but counter to past studies of library voting, these data show an approximate 2% decrease in voter support for each one percentage point increase in portion of Republican voters in a precinct. It is also distinct in magnitude from the analysis of national data.

Environmental conditions, like extreme heat and air quality measures, are not used as covariates in published studies of voter behavior. Ethnographic and qualitative studies of library responses to disaster, including extreme heat events, exist, but no quantitative analysis of library service provision, use, or outcomes relationship, includes environmental covariates in their models. In studies of how voters respond to hazards, people show increased fiscal conservatism with increased perceptions of risk [49].

As noted, library service and use statistics are not captured at the building level for the bulk of the study years. Within a vote event, there is no variation in library service across the precincts, except in distance to service. When viewed in relationship with distance to outlets and whether the precinct’s nearest library is from the same system as the one asking for more money, a story of proximity emerges. The impact of every additional mile to the library (Model 6) is nearly 20 times more negative than every additional dollar in cost.

### Willingness to Pay (WTP) results

Given the log-odds analysis, annual household WTP calculations for both the library system overall, and the nearness of library service (in *DIST_MILES*) are provided. Proximity to library outlet is an important determinant of voter behavior. We explore what this means for WTP, following Anderson, et al. [48], where the decomposition of WTP into its components is done by dividing the estimated coefficient on the specific component by $\hat{\alpha }$.

Table F5 in Appendix F shows the calculation results for library systems where the precinct level vote shares varied enough that robust estimates converged with a strongly restricted sample. Library system mean household WTP was calculated as both the direct calculation from equation (10) above, as the division of precinct log-odds by system-wide regression $\hat{\alpha }$, and as a mean weighted by the total number of votes cast within each precinct. As expected, the lower the total number of votes cast, the less variation between WTP estimations.

Point estimates of annual household WTP range from $25 to $65, expanding to $4 to $99 when incorporating the outer limits of the standard deviations. The decomposition of value for distance to the nearest library is not weighted because it doesn’t incorporate precinct level information directly but instead is a ratio of maximized estimates from the overall general linear model.

### Distance decay of value

Because decomposition of WTP for library distance is in relationship to overall WTP for the public library system, it’s value can be seen first in the scale difference between the coefficient point estimates, with highest cost of distance attributed when the coefficient on DIST_MILES is 27 times higher than that on TAX_INCID. Overall, the range of statistically significant point estimates shows that voters are 3% to 8% less likely to vote Yes for every mile of distance between their precinct and the closest library building, as shown in the widening margins of error in Fig 3 below. Consistently, the overall model showed a 4% per mile effect and shows the robust importance of public library as built environment. In the data with both statistically significant findings and a large number of precincts, voters lose $33 in annual household library value per mile of distance.

**Fig 3:**
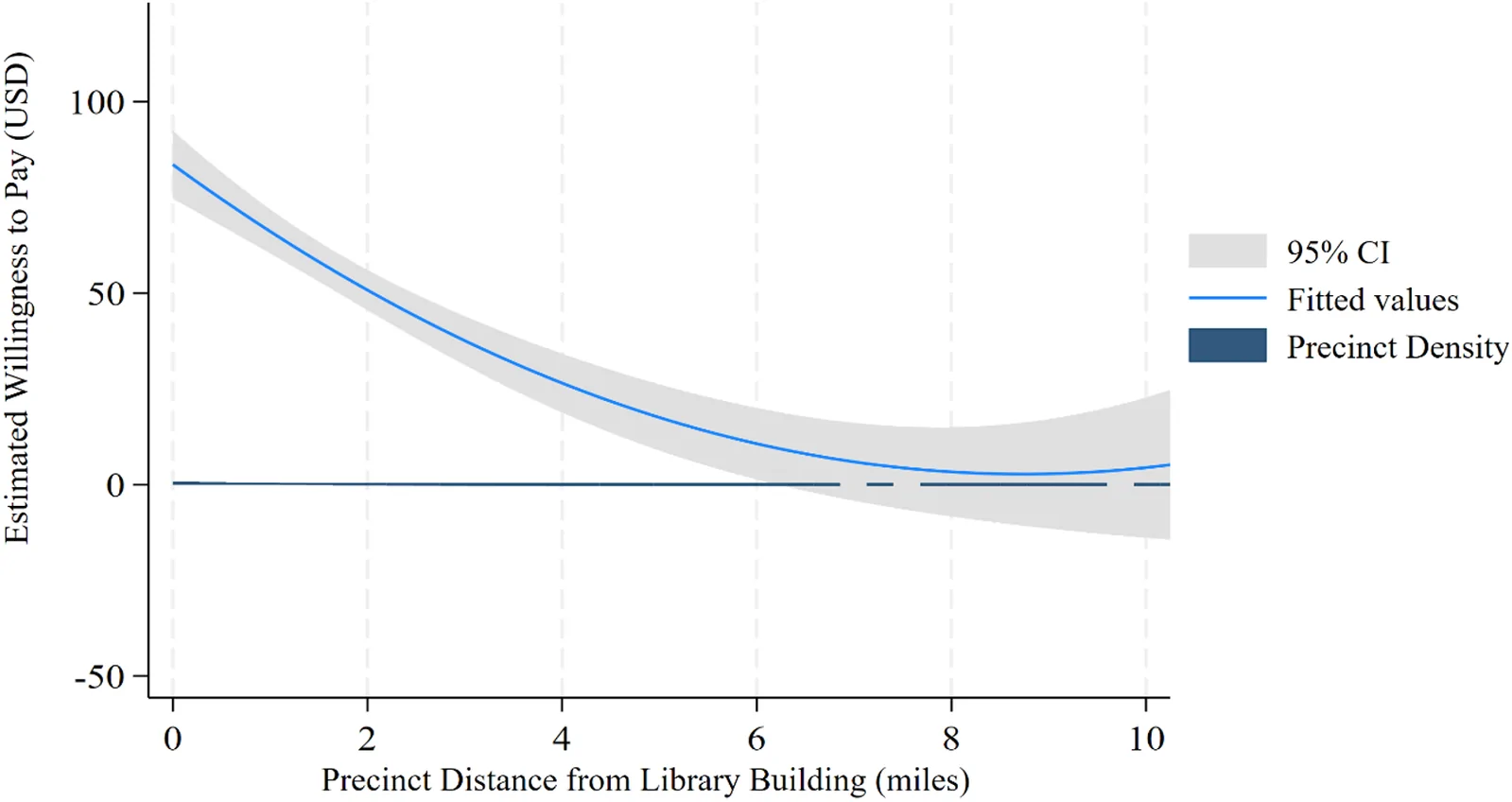
Estimated WTP Decay with Distance to a Public Library. *Sources*. Author’s own figure from Washington Referendum Data 2010–2022, restricted to library systems 90053, 15003, and 350060. *Notes*. What appears to be a dashed line at zero shows the density of precincts at any interval of distance in miles from a library. As the density of voting precincts diminishes, the width of error margins increases, with reliability in estimation ceasing around the 8 mile mark. Only precincts with overall significant results are shown.

Voter behavior in library funding referendums is more significantly predicted by exogenous factors than specific services. Nationally, this means that the institutional context for the vote—state, year, level of rurality, and administrative structure (including service geography and single or multiple outlet)—together accounts for 64% of the variation in the probability of a Yes. A similar pattern emerges for Washington state in precinct level analysis, but to a lesser degree. Finer grain data allows additional effects, like partisanship and distance to nearest library, to emerge. At both levels of analysis, the prevalence of extreme heat-days shows a probabilistic correlation with voter preferences for library funding, but in opposite directions.

## Discussion

The objective of this analysis was to understand determinants of voter behavior toward public libraries in funding referendums, and how environmental conditions, like geography and climate change-driven hazards, influence voting. This study used two novel datasets of library referendum events, one national library system vote-year, and another precinct vote-year within Washington state. General linear modeling was employed on a binomial distributed proportion of Yes votes at either the vote event or precinct level, estimated with a logit linked maximum likelihood function. Voters overwhelmingly support libraries with the cost (tax incidence) of the support showing less importance, in magnitude or significance, than factors like local Republican vote share, educational attainment, or housing cost burden. Where proximity was known, having a public library building within the vote district was the single most important determinant.

Public libraries are uniquely valuable assets to their communities. Although other semi-public use amenities are voted on, like greens spaces and parks [20], and public schools, neither is substitutable with libraries. It is the unique availability to every person in the geographic zone, its structural sheltering, and its socio-civic functions that set libraries apart among common public goods in the US. In that relationship between the built environment and social amenity, data availability really only allows for measuring the relationship between the building characteristics and its covariates. Placemaking valuation is confounded by lack of geographically precise service and usage measurements. Even given such limitations, spatial analysis provides insights into the importance of library access.

Understanding how much of this importance is driven by the unique institutional and environmental contexts of Washington state is a necessary next step. As noted earlier, rising in-migration of higher-income households into rural Washington raises assessed property values, and thus both funding levels and, mechanically, tax incidence estimates, which may compress the observed price effect and inflate apparent library support relative to a lower-growth state. While we believe that findings on distance and facility access are less likely to be confounded, since they are structural and spatial rather than income-driven, it is feasible that findings involving wealth-related covariates could plausibly be conditioned on Washington’s specific and dynamic housing market, rather than as representative of a “typical” state. Therefore, additional state-based studies that replicate the present methods are called for in states with high frequency referendums where precinct-level data retrieval is more challenging.

It’s evident from the national study of library voting that state contexts have outsized impacts on the structure of the ballots themselves, but also on norms around voting and public funding. Public libraries enjoy majority favor in a near unanimity of the referendums, but variation between precincts could help library service providers as well as advocates gain clarity on future challenges. State-level institutional heterogeneity means the national-level results describe average tendencies across highly varied institutional contexts.

The library system level household WTP for service in the Washington data should be seen as an initial effort, with two point-estimates obtained from only 19 full information vote events. Washington case study results are conditioned on Washington’s specific institutional and demographic context rather than generalizable to each library system in Washington or nationally. Further state-level case studies may allow enough robust estimates that service-based determinates could be analyzed. We hope this analysis spurs further investigation.

Finally, environmental conditions have a role in community needs that should be incorporated into empirical analyses of public libraries. This study is the first to model environmental conditions as a significant influence in library voting. Results provide a cautionary tale to publicly provisioned service organizations in an era of increasing severity and frequency of weather (e.g., extreme heat days), air pollutants and wildfire-smoke hazards [63,64]. Resilience researchers have examined conditions necessary for community responses to disasters [7]. Public libraries are well distributed geographically and positioned to provide structural (informational, social, organizational, and sheltering) support to communities.

## Supporting information

S1 File Appendix Voters Reveal Library Preference. (DOCX)
