# Supplementary material for: Voters reveal public library value: Evidence from US referendums
Source: PLoS One. 2026 Oct 1;21(10):e0351352. doi: 10.1371/journal.pone.0351352 (PMC13630217; doi:10.1371/journal.pone.0351352)
Supplement: Supplementary file 1 [file pone.0351352.s001.docx]

# Voters Reveal Library Preference

## Appendix A Public Library Geography of Institutional Arrangements

Figure A1. State policy context impacts vote frequency, approval, and funding

14 – 559

5 – 14

2 – 5

1 – 2

Count of Vote Events

2010 – 2021

|  |  |
| --- | --- |
| State Average Percent Yes  2010 – 2021  67% - 84%  62% - 67%  58% - 62%  32% - 58% |  |
| Average Annual Household Tax  Incidence  $638 - $1,387  $179 - $638  $99 - $179  $11 - $99  No data |  |
| Average Annual Local Funding Per Capita ($)  $51 - $88  $42 - $51  $26 - $42  $7 - $26 |  |

Source. Library data from: Public Library Survey (IMLS), EveryLibrary, Library Journal, State Library Agencies of California, Michigan, New York, and Ohio, Ballotpedia, and library workers in California, Illinois, Iowa, Maryland, Nebraska, New York, South Carolina, Texas, Utah, and Wyoming. State boundary data from: U.S. Census Bureau, “tl_2021_us_state”, TIGER/Line Shapefiles, 2021, https://www2.census.gov/geo/tiger/TIGER2021/STATE/, accessed 2023. Author’s own figure generated in Stata16 using spmap.

(51 – 88)

(42 – 51)

(26 – 42)

(7 – 26)

(638 – 1,387)

(179 – 638)

(99 – 179)

(11 – 99)

Note. Ohio has what appears to be a conflict between high tax incidence and low per capita local funding. That is correct. Ohio has a statewide public library funds that channels 1.7% of all sales taxes collected in the state to public libraries.

Figure A2: Library Systems with County Based Service Geographies

Source. Library data from: Public Library Survey (IMLS), 2021 Reporting Year, accessed 2023. State and county boundary data from U.S. Census Bureau, “tl_2021_us_state”, TIGER/Line Shapefiles, 2021, https://www2.census.gov/geo/tiger/TIGER2021/STATE/, accessed 2023. Author’s own figure generated in Esri ArcGIS.Author’s own figure.

Note. Color coding to obviate distinct library systems but has no other meaning. Large geographies represent multiple-county service boundaries. Where a system is a City-County combined district, the county boundary is displayed. Like the regionality of institutional arrangements, library service boundaries are based in the history of local library development.

Figure A3. Heterogeneity of Public Library Administrative Structure

Source. Library location data from Public Library Survey (IMLS), 2021 Reporting Year, accessed 2023.; State boundary data from U.S. Census Bureau, “tl_2021_us_state”, TIGER/Line Shapefiles, 2021, https://www2.census.gov/geo/tiger/TIGER2021/STATE/, accessed 2023. Author’s own figure generated in Esri ArcGIS.

Note. The governance and administrative structure, as well as whether a library is single or multi-outlet, is driven in large part by state level policies and regional histories. In the Northeast, many libraries were formed out of private book clubs and ladies’ associations, which persists as independent non-profits represented in orange. Throughout the north, municipal or village culture is the primary policy driver and historic recipient of Carnegie Grants for the construction of public libraries in the early 20^th^ century, represented in dark blue. Most of the US, geographically, is made up of library systems with multiple facilities (branches) with a main or central location. Each of these branches is represented by a light grey triangle and the central branch indicates the jurisdiction coverage type. The south, apart from clusters in Appalachia and the rural Midwest, is dominated by county (light green) or multi-county (lavender) library systems. School district (bright pink) and tribal nation administered (red), are also grounded in the institutional history of their respective regions.

## Appendix B. Data Sources

#### Table B1. Data Sources

| National | | |
| --- | --- | --- |
| Concept | Source | Variables |
| Identifier | IMLS Library Search & Compare; State Library agencies | fscskey, libkey |
| Votes | EveryLibrary, Library Journal, State Library Agencies, library worker survey | (22 variables) time variables, tax amounts and calculations, vote amounts and calculations, dummy variables for factors including vote month and type |
| Library System information | IMLS Public Library Survey | (64 variables) all library system administrative, funding, geographic, and service characteristics |
| Extreme Heat | CDC National Environmental Public Health Tracking Network (Accessed From: https://ephtracking.cdc.gov/DataExplorer. Accessed on 05/02/2024) | (8 variables) count variables of days either the heat index or unadjusted raw temperature exceeded a threshold every 5 degrees from 90-105 F |
| Resident characteristics | Census American Community Survey | (14 variables) median house value, median household income, unemployment, poverty, number of individuals in household and of households, portion of households with children or elders, educational attainment, population stability, portion of population born outside the US |
| Tax Incidence | Calculated from vote information | tax_incid and lntax are calculated from the levy rate multiplied by median household value divided by 1,000 and then the log transformation of the calculation result |
| Geography | IMLS Public Library Survey, Census TIGER shape files | (7 variables) county and state FIPS as string and numeric, Census tract id, NCIS LEA (school district) id |
| Partisanship | MIT Election Lab | thirdparty and republican are the respective portions of voters who cast ballots toward those presidential candidates in 2012,2016, and 2020 |
| Risk & Resilience | FEMA National Risk Index | (7 variables) raw values and scores for damages, risk, and resiliences indexes' |
| Air Quality | EPA Outdoor Air Quality Data | (3 variables) count variables of days with low AQI, high AQI, and high PM2.5 concentrations |
| Washington | | |
| Concept | Source | Variables |
| Identifier | Generated, Washington State Library, Library Journal | event_id, fscskey (string and byte), libraryssytem |
| Votes | Washington State Library, Library Journal, calculated | (32 variables) time variables, tax amounts and calculations, vote amounts and calculations, dummy variables for factors including vote month and type |
| Geography | County Election Board website, Secretary of State Precinct boundary files, Census TIGER Census boundary files, generated transformations | (11 variables) precinct names and codes matched to overlapping Census counties, tracts, and block groups |
| Resident characteristics | Census American Community Survey, Secretary of State Precinct boundary files, Census TIGER Census boundary files | (31 variables) all household value, income, size, race and ethnicity, educational attainment, and composition variables and calculated percentages from raw counts |
| Nearest library service | Calculated in ArcGIS: Washington State Library outlet location file XY point count within precinct polygon or distance to centroid | (7 variables) binary of outlet in precinct, distance to centroid, outlet identifier, square footage, hours, and weeks open in a year |
| Extreme Heat | CDC National Environmental Public Health Tracking Network (Accessed From: https://ephtracking.cdc.gov/DataExplorer. Accessed on 05/02/2024) | (8 variables) count variables of days either the heat index or unadjusted raw temperature exceeded a threshold every 5 degrees from 90-105 F |
| Risk & Resilience | FEMA National Risk Index | (8 variables) raw values and scores for damages, risk, and resiliences indexes with tag for county or tract match |
| Tax Incidence | Calculated from vote information | (3 variables) tax_incid is calculated from the levy rate multiplied by median household value divided by 1,000; bondtax_estimate is total bond/count of houses= x, x/total bond term. Lntax is the log tranformation of these combined variables. |
| Partisanship | Secretary of State - presidential election results by precinct | (4 variables) portion of voters choosing a republican presidential candidate in 2012, 2016, or 2020 and an unweighted average of those portions. |
| Air Quality | EPA Outdoor Air Quality Data | (3 variables) count variables of days with low AQI, high AQI, and high PM2.5 concentrations |

## Appendix C. Voter Characteristics & Vote Event Tables

#### Figure C1. Income Distribution by Voted Status Reported in Current Population Survey

Weekly Earnings in US Dollars

Source. Data extracted from the IPUMS CPS, a time and population harmonized version of the US Census Bureau and Bureau of Labor Statistics Current Population Survey, bi-annual waves 2010-2022 (Flood et al., 2024). Graphs are generated by the author.

#### Table C1. Votes, Taxes, and Yes Percentages by State

| State | Vote Events | Operating Tax Incidence | Bond Tax Incidence | % Yes Votes |
| --- | --- | --- | --- | --- |
| New York | 559 | $ 150.4 | $ 1,408.2 | 76% |
| Ohio | 412 | $ 195.2 | $ 263.8 | 65% |
| Michigan | 385 | $ 107.3 | $ 112.3 | 64% |
| Washington | 43 | $ 89.3 | $ 125.4 | 60% |
| Illinois | 38 | $ 514.8 | $ 290.8 | 50% |
| Colorado | 31 | $ 741.4 | $ 42.5 | 57% |
| Louisiana | 21 | $ 848.5 | $ 2,955.8 | 62% |
| California | 15 | $ 13.5 |  | 72% |
| Oregon | 13 | $ 125.5 | $ 764.7 | 55% |
| Idaho | 12 | $ 332.0 | $ 2,266.7 | 58% |
| Montana | 12 | $ 795.6 |  | 58% |
| Texas | 11 | $ 347.8 |  | 66% |
| Connecticut | 9 | $ 567.6 | $ 517.3 | 54% |
| Massachusetts | 9 | $ 1,629.7 | $ 982.0 | 67% |
| New Hampshire | 9 | $ 22.7 |  | 61% |
| Iowa | 7 | $ 103.9 |  | 64% |
| Maryland | 6 | $ 72.9 |  | 84% |
| Pennsylvania | 6 | $ 12.6 |  | 57% |
| Nebraska | 5 | $ 0.5 | $ 484.0 | 55% |
| Alaska | 4 | $ 4.3 | $ 3.5 | 61% |
| South Carolina | 4 | $ 283.0 |  | 67% |
| Wisconsin | 4 |  | $ 750.4 | 59% |
| Maine | 3 | $ 39.7 |  | 58% |
| Utah | 3 | $ 76.2 |  | 44% |
| Arkansas | 2 | $ 146.7 |  | 65% |
| Florida | 2 | $ 42.3 | $ 725.0 | 59% |
| West Virginia | 2 | $ 10.8 |  | 70% |
| Nevada | 2 | $ 169.0 | $ 1,215.9 | 32% |
| New Jersey | 1 | $ 113.1 |  | 50% |
| Rhode Island | 1 |  | $ 357.7 | 72% |
| Virginia | 1 |  | $ 75.6 | 65% |

*Sources*. Public Library Survey (IMLS), EveryLibrary, Library Journal, State Library Agencies of California, Michigan, New York, and Ohio, Ballotpedia, and library workers in California, Illinois, Iowa, Maryland, Nebraska, New York, South Carolina, Texas, Utah, and Wyoming.

*Notes*. During the window 2010 – 2022, 8 states had no public votes on library administration or funding. Hawaii’s public libraries are operated by the state library agency with unique, non-local funding and administrative structure. Similarly, Washington, DC libraries are one system that, like many of the nation’s largest urban libraries, does not put funding on the ballot. 16 states had referendums of some kind, including annexation, establishment, or other administrative actions, with no tax amount published. High tax incidence is related to high median house value, and low time-period terms. All of these are controlled within estimation models. Full list of vote events by state in Appendix A.1 and complete table of summary statistics for the national vote data is given in Appendix A.

#### **Table C2.** Precincts, Votes, Taxes, and Yes Percentages by County (WA)

| County | Precincts | Average Total Votes per Precinct | Operating Tax Incidence | Percent Yes Votes |
| --- | --- | --- | --- | --- |
| Benton | 17 | 417 | $ 100.9 | 52% |
| Clallam | 95 | 255 | $ 40.5 | 60% |
| Clark | 187 | 420 | $ 137.5 | 49% |
| Cowlitz | 18 | 378 | $ 89.5 | 60% |
| Grays Harbor | 11 | 536 | $ 17.7 | 55% |
| Island | 47 | 449 | $ 175.4 | 60% |
| King | 3,332 | 173 | $ 137.1 | 62% |
| Kitsap | 196 | 309 | $ 24.8 | 64% |
| Klickitat | 29 | 186 | $ 93.4 | 60% |
| Pend Oreille | 27 | 133 | $ 24.6 | 63% |
| Pierce | 360 | 568 | $ 166.0 | 50% |
| San Juan | 13 | 596 | $ 76.6 | 60% |
| Skamania | 25 | 116 | $ 132.2 | 52% |
| Snohomish | 649 | 152 | $ 201.1 | 48% |
| Spokane | 644 | 312 | $ 74.4 | 60% |
| Stevens | 131 | 299 | $ 53.5 | 45% |
| Whatcom | 1 | 752 | $ 125.9 | 55% |
| Whitman | 39 | 109 | $ 79.8 | 73% |

*Sources*: Washington Operating Votes dataset 2010 through2022.

*Notes*: Only operational funding votes are included in the data, which narrows the tax incidence spread.

## Appendix D. Codebooks & Summary Statistics

#### Table D1. National Vote Dataset Codebook & Summary Statistics

| Variable | Label | Obs | Unique | Mean | Std. dev. | Min | Max |
| --- | --- | --- | --- | --- | --- | --- | --- |
| FSCSKEY | Federal library system identifier | 1,679 | 802 | . |  | . | . |
| LIBKEY | Numeric library system identifier constructed from FSCSKey and FIPS code | 1,679 | 802 |  |  |  |  |
| YEAR | Calendar year of vote event | 1,679 | 12 | 2015.64 | 3.31 | 2010 | 2021 |
| STATE | State of library location | 1,679 | 41 | . |  | . | . |
| NUMSTATE | Numeric FIPS/INCITS code for state with library system | 1,678 | 41 | 33 | 9 | 2 | 56 |
| COUNTY | County of library administrative center location | 1,679 | 332 | . |  | . | . |
| NUMCOUNTY | 5 digit code for state and county | 1,679 | 379 | 33234 | 8963 | 2020 | 56021 |
| MONTH | Month of vote event | 1,314 | 14 | . |  | . | . |
| TYPE | Vote type: operating, operating renewal, renewal with increase, capital bond | 1,186 | 64 | . |  | . | . |
| BOND | 1=referendum is on a capital bond; 0=all other referendum types | 1,679 | 2 | 0.03 | 0.16 | 0.00 | 1.00 |
| MILLAGE | Millage rate on ballot | 874 | 208 | 1.17 | 1.10 | 0.02 | 11.10 |
| NEW_MILL | New millage rate if specified on ballot | 121 | 61 | 0.80 | 0.98 | -0.15 | 8.50 |
| CUR_MILL | Current millage rate if specified on ballot | 28 | 27 | 3.69 | 2.41 | 0.75 | 11.10 |
| TOT_MILL | Total millage rate if specified on ballot | 521 | 145 | 1.41 | 1.33 | 0.05 | 11.10 |
| NEW_LEVY | New levy rate if specified on ballot | 3 | 3 | 0.17 | 0.10 | 0.07 | 0.27 |
| CUR_LEVY | Current levy rate if specified on ballot | 3 | 3 | 30.83 | 52.98 | 0.15 | 92.00 |
| TOT_LEVY | Total levy rate if specified on ballot | 4 | 4 | 24.32 | 47.87 | 0.22 | 96.12 |
| TOT_TAX | Tax rate on ballot | 23 | 18 | 14.38 | 23.47 | 0.00 | 75.00 |
| NEW_TAX | New tax rate if specified on ballot | 7 | 5 | 0.00 | 0.01 | 0.00 | 0.02 |
| CUR_TAX | Current tax rate if specified on ballot | 2 | 2 | 0.01 | 0.01 | 0.00 | 0.01 |
| NEW_FUNDS | New funding amount ($) if specified on ballot, or reported to state | 412 | 216 | $1,350,132 | $5,636,035 | $ - | $60,800,000 |
| CUR_FUNDS | Current funding amount ($) if specified on ballot, or reported to state | 258 | 175 | $ 496,099 | $ 843,186 | $ - | $ 5,656,000 |
| TOT_FUNDS | Total funding amount specified on ballot or reported to state | 357 | 261 | $4,812,461 | $25,100,000 | $ - | $387,000,000 |
| TAXINCID_TOTAL | Tax incidence based on total millages, levies, or funds | 860 | 799 | 206.57 | 1,255.43 | 0.00 | 35,958.49 |
| TAXINCID_NEW | Tax incidence based on new millages, levies, or funds | 537 | 510 | 86.81 | 292.03 | -38.37 | 3,013.52 |
| TAXINCID_MIXED | Tax incidence combination of total and new, where appropriate | 1,136 | 1,110 | 200.70 | 1,119.22 | -29.21 | 35,958.49 |
| FOR | Votes for referendum | 1,533 | 1,205 | 6,384.53 | 25,315.79 | 19.00 | 450,514.00 |
| AGAINST | Votes against referendum | 1,533 | 1,059 | 3,158.86 | 9,962.88 | 0.00 | 150,652.00 |
| TOTALVOTES | Total votes cast in library referendum | 1,533 | 1,267 | 9,543.39 | 33,398.43 | 19.00 | 450,541.60 |
| YES_PERC | Percent votes for referendum | 1,679 | 1,532 | 0.67 | 0.13 | 0.14 | 1.00 |
| MAJORITY | Binary: 1= 50% or more yes votes | 1,679 | 2 | 0.91 | 0.29 | 0.00 | 1.00 |
| ODDSYES | Odds ratio calculated as %yes / %no | 1,667 | 1,531 | 3.08 | 4.20 | 0.17 | 86.00 |
| LGODDS | Log transformation of the odds ratio where negative numbers are less than 50% yes votes | 1,667 | 1,531 | 0.80 | 0.71 | -1.79 | 4.45 |
| C_LEGBAS | Legal basis or governance structure of system: municipal, county, non-profit agency | 1,679 | 8 | . |  | . | . |
| SERVICE_GEO | Numeric transformation c_legbas | 1,679 | 8 | 4.47 | 2.21 | 1.00 | 9.00 |
| C_FSCS | Federal definition of a library met: Y for yes, N for no | 1,679 | 2 | . |  | . | . |
| FED_LIBRARY | Numeric transformation c_fscs: 1=Yes, meets federal definition, 0=No | 1,679 | 2 | 1.00 | 0.04 | 0.00 | 1.00 |
| POPU_LSA | Population served by library | 1,678 | 1,027 | 54,704.50 | 171,594.80 | 210.00 | 3,810,129.00 |
| POPU_UND | Population served by library less populations in overlapping service areas | 1,678 | 1,547 | 54,405.15 | 170,791.70 | 208.00 | 3,792,153.00 |
| CNTYPOP | Population of county where library system administration is located | 1,678 | 1,049 | 312,477 | 635,560 | 6,007 | 10,200,000 |
| LOCALE | Locale code assigned to library administration location: 11=urban, 21=suburban, 31=small town, 41=rural | 1,678 | 12 | 30 | 10 | 11 | 43 |
| DENSITY | Based on locale code: 1=Urban, 2=Suburban, 3=Small Town, 4=Rural | 1,678 | 4 | 2.79 | 0.97 | 1.00 | 4.00 |
| CENTLIB | Count of central libraries within library system | 1,678 | 2 | 0.96 | 0.20 | 0.00 | 1.00 |
| BRANLIB | Count of branch libraries within library system | 1,678 | 33 | 1.66 | 4.86 | 0.00 | 72.00 |
| BKMOB | Count of bookmobiles operated by library system | 1,678 | 7 | 0.12 | 0.49 | 0.00 | 8.00 |
| SINGMULT | 1= single outlet library system; 2 = multiple outlet library system | 1,678 | 2 | 1.26 | 0.44 | 1.00 | 2.00 |
| TOTOUT | Count of total outlets in library system | 1,678 | 32 | 2.62 | 4.79 | 1.00 | 73.00 |
| AVG_SQFT | Average square footage within library system | 1,678 | 780 | 12,609 | 12,881 | 432 | 124,000 |
| TOT_SQFT | Total square footage of all outlets within library system | 1,678 | 779 | 37,612 | 100,215 | 432 | 1,391,789 |
| MASTER | Count of master's degree holding librarians (FTE) | 1,678 | 422 | 6.98 | 19.30 | 0.00 | 337.00 |
| LIBRARIA | Count of staff with "librarian" title (FTE) | 1,678 | 621 | 9.67 | 23.24 | 0.00 | 337.00 |
| OTHPAID | Count of other paid staff (FTE) | 1,678 | 878 | 19.75 | 54.32 | 0.00 | 600.00 |
| TOTSTAFF | Total paid staff (FTE) | 1,678 | 1,083 | 29.42 | 74.75 | 0.00 | 937.00 |
| STFHRS | Total annual staff hours summed across all outlets calculated as: TOTSTAF * 2080 | 1,678 | 1,083 | 61,187 | 155,490 | 0 | 1,948,960 |
| LOCGVT | Non-capital funding ($) from local gvt sources | 1,678 | 1,563 | $2,147,306 | $7,002,294 | $- | $125,000,000 |
| FUNDCAPITA | Local funding per capita calculated: LOCGVT / POP_LSA | 1,678 | 1,567 | $ 40.15 | $ 36.44 | $ - | $ 432.16 |
| STGVT | Non-capital funding ($) from state gvt sources | 1,678 | 1,444 | $ 437,682 | $2,007,986 | $ - | $ 39,800,000 |
| FEDGVT | Non-capital funding ($) from federal gvt sources | 1,678 | 261 | $ 7,571 | $ 50,654 | $ - | $ 1,105,126 |
| OTHINCM | Non-capital funding ($) from non-gvt sources | 1,678 | 1,618 | $ 178,270 | $598,608 | $ - | $ 10,200,000 |
| TOTINCM | Total non-capital revenues ($) | 1,678 | 1,677 | $2,770,828 | $8,128,473 | $11,258 | $128,000,000 |
| PRMATEXP | Print material expenditures ($) | 1,678 | 1,659 | $ 149,202 | $ 428,163 | $ - | $ 7,352,277 |
| ELMATEXP | Digital material expenditures ($) | 1,678 | 1,217 | $ 89,798 | $372,085 | $- | $ 5,435,556 |
| OTHMATEX | Other, A/V material expenditures ($) | 1,678 | 1,531 | $ 71,187 | $223,641 | $ - | $ 3,063,417 |
| TOTEXPCO | Total collection expenditures ($) | 1,678 | 1,667 | $ 310,186 | $952,968 | $- | $ 11,900,000 |
| TOTOPEXP | Total operating expenditures ($) | 1,678 | 1,677 | $2,578,511 | $7,725,238 | $12,228 | $113,000,000 |
| LCAP_REV | Capital funding ($) from local government | 1,678 | 170 | $ 62,237 | $ 674,367 | $ - | $ 21,000,000 |
| SCAP_REV | Capital funding ($) from state government | 1,678 | 142 | $ 5,301 | $38,296 | $ - | $ 738,000 |
| OCAP_REV | Capital funding ($) from non-government sources | 1,678 | 446 | $ 32,458 | $269,480 | $ - | $ 5,780,000 |
| CAP_REV | Total capital funding ($) | 1,678 | 635 | $ 103,361 | $ 733,217 | $ - | $ 21,000,000 |
| CAPITAL | Total capital expenditures ($) | 1,678 | 1,043 | $ 163,202 | $ 738,738 | $ - | $ 18,500,000 |
| BKVOL | Total print books in collection | 1,678 | 1,666 | 133,025 | 391,312 | 1,946 | 6,459,552 |
| EBOOK | Total e-books in collection | 1,678 | 979 | 79,104 | 197,674 | 0 | 5,192,359 |
| AUDIO_PH | Total physical audio items in collection | 1,678 | 1,495 | 9,660 | 25,514 | 0 | 437,113 |
| AUDIO_DL | Total digital audio items in collection | 1,674 | 921 | 44,231 | 291,539 | 0 | 11,300,000 |
| VIDEO_PH | Total physical video items in collection | 1,678 | 1,581 | 13,875 | 34,171 | 0 | 543,932 |
| VIDEO_DL | Total digital video items in collection | 1,672 | 481 | 5,272 | 38,483 | 0 | 915,760 |
| DATABASE | Count of databases available | 622 | 142 | 96 | 121 | 0 | 771 |
| TOTCOLL | Total of all physical items held within library systems across all outlets | 1,678 | 1,668 | 156,560 | 442,834 | 2,097 | 6,986,141 |
| HRS_OPEN | Open hours summed across all library outlets annually | 1,672 | 930 | 6,085 | 11,742 | 0 | 151,632 |
| VISITS | Visits to libraries summed across all library outlets annually | 1,678 | 1,634 | 261,257 | 742,645 | 0 | 12,500,000 |
| REFERENC | Reference transactions summed across all library outlets annually | 1,678 | 1,451 | 47,523 | 159,311 | 0 | 3,456,300 |
| REGBOR | Count of library card holders | 1,678 | 1,613 | 31,388 | 91,623 | 56 | 1,306,822 |
| TOTCIR | Total items borrowed digitally and physically, summed across all library outlets annually | 1,678 | 1,672 | 520,627 | 1,604,842 | 184 | 22,400,000 |
| KIDCIRCL | Total children's items borrowed summed across all library outlets annually | 1,678 | 1,654 | 161,060 | 481,410 | 0 | 5,376,237 |
| LOANTO | Total physical items lent to a library in a different library system, annually | 1,678 | 1,520 | 19,707 | 55,126 | 0 | 1,059,499 |
| LOANFM | Total physical items borrowed to a library in a different library system, annually | 1,678 | 1,565 | 19,330 | 49,987 | 0 | 970,559 |
| TOTPRO | Total program instances hosted summed across all library outlets annually | 1,677 | 955 | 921 | 2,139 | 0 | 24,924 |
| KIDPRO | Total children's program instances hosted summed across all library outlets annually | 1,677 | 718 | 521 | 1,284 | 0 | 14,324 |
| YAPRO | Total teen or young adult program instances hosted summed across all library outlets annually | 1,674 | 321 | 86 | 235 | 0 | 3,785 |
| TOTATTEN | Total program attendees summed across all library outlets annually | 1,677 | 1,606 | 20,269 | 49,309 | 0 | 496,219 |
| KIDATTEN | Total children's program attendees summed across all library outlets annually | 1,677 | 1,535 | 13,447 | 34,285 | 0 | 405,070 |
| YAATTEN | Total teen or young adult program attendees summed across all library outlets annually | 1,674 | 893 | 1,431 | 4,991 | 0 | 92,157 |
| GPTERMS | Total public access computer stations summed across all library outlets annually | 1,677 | 219 | 52 | 154 | 1 | 3,801 |
| PITUSR | Total uses of public access computer stations summed across all library outlets annually | 1,678 | 1,576 | 54,026 | 194,876 | 0 | 3,880,000 |
| COMPUSE | Total uses of library provided internet, either via computing station or wi-fi connection, calculated: pitusr + wifisess | 1,678 | 1,626 | 108,790 | 821,710 | 0 | 31,400,000 |
| HOUSES | Count of houses | 1,678 | 1,644 | 68,935 | 352,958 | 123 | 7,775,774 |
| HOUSEVALE | Median house value estimate | 1,678 | 1,217 | $ 181,665 | $ 117,360 | $17,200 | $ 971,000 |
| HOUSEVALM | Median house value margin of error | 1,678 | 1,586 | $ 19,614 | $ 26,845 | $553 | $ 697,680 |
| MHHIE | Median household income estimate | 1,678 | 1,578 | $ 55,001 | $21,584 | $9,935 | $ 180,455 |
| MHHIM | Median household income margin of error | 1,678 | 1,529 | $ 8,398 | $ 5,602 | $428 | $ 52,495 |
| UNEMPLPE | Unemployment rate estimate | 1,678 | 216 | 7.92 | 4.75 | 0.00 | 47.90 |
| POVPE | Poverty rate estimate | 1,678 | 322 | 10.58 | 9.00 | 0.00 | 67.40 |
| HHSIZE | Average household size estimate | 1,678 | 187 | 2.43 | 0.32 | 1.14 | 4.03 |
| KIDHHPE | Percent households with children | 1,678 | 374 | 28.68 | 8.26 | 0.00 | 64.70 |
| JUSTHSPE | Percent population high school education or equivalence as highest education | 1,678 | 425 | 32.95 | 10.46 | 3.60 | 100.00 |
| BAPLUSPE | Percent population with a bachelor's degree or more education | 1,678 | 524 | 26.61 | 14.78 | 0.00 | 81.80 |
| STABLEPE | Percent residents who lived in the same house last year | 1,678 | 346 | 85.13 | 8.61 | 38.30 | 100.00 |
| FORBORNPE | Percent residents who were not born in the US or US territories | 1,678 | 231 | 5.32 | 6.36 | 0.00 | 59.40 |
| ELDHHPE | Percent households with individuals over age 65 living alone | 1,678 | 598 | 12.33 | 5.21 | 0.42 | 38.60 |
| THIRDPARTY | Average third party candidate share of presidential elections | 1,673 | 713 | 0.04 | 0.03 | 0.01 | 0.35 |
| AVGREP | Average republican candidate share of presidential elections (2012,2016,2020) | 1,675 | 1,025 | 50.59 | 11.69 | 9.10 | 88.32 |
| PRES_YEAR | 1=presidential election year | 1,679 | 2 | 0.26 | 0.44 | 0.00 | 1.00 |
| INDEX100 | Annual total days heat index 100 F degrees + | 1,674 | 57 | 5.06 | 9.74 | 0.00 | 87.00 |
| INDEX105 | Annual total days heat index 105 F degrees + | 1,674 | 32 | 1.45 | 3.70 | 0.00 | 36.00 |
| INDEX90 | Annual total days heat index 90 F degrees + | 1,674 | 111 | 25.31 | 23.63 | 0.00 | 165.00 |
| INDEX95 | Annual total days heat index 95 F degrees + | 1,674 | 86 | 12.53 | 16.90 | 0.00 | 130.00 |
| TEMP100 | Annual total days abs. temp 100 F degrees + | 1,674 | 27 | 0.66 | 4.74 | 0.00 | 97.00 |
| TEMP105 | Annual total days abs. temp 105 F degrees + | 1,674 | 13 | 0.14 | 1.79 | 0.00 | 55.00 |
| TEMP90 | Annual total days abs. temp 90 F degrees + | 1,674 | 87 | 10.60 | 17.26 | 0.00 | 149.00 |
| TEMP95 | Annual total days abs. temp 95 F degrees + | 1,674 | 49 | 2.60 | 9.25 | 0.00 | 128.00 |
| RISK_VALUE | National Risk Index value | 1,679 | 379 | 44,100,000 | 217,000,000 | 209,390 | 5,330,000,000 |
| RISK_SCORE | National Risk Index relative score | 1,679 | 379 | 60.63 | 26.99 | 0.48 | 100.00 |
| RESL_SCORE | NRI Community Resilience relative score | 1,679 | 379 | 76.60 | 17.02 | 8.91 | 99.87 |
| RESL_VALUE | NRI Community Resilience index value | 1,679 | 379 | 2.71 | 0.08 | 2.41 | 2.96 |
| CRF_VALUE | NRI Community Resilience Factor, a value take from USC BRICS | 1,679 | 379 | 1.07 | 0.11 | 0.77 | 1.45 |
| GOODDAYS | Annual count of low AQI days | 1,022 | 241 | 236.58 | 80.86 | 2.00 | 356.00 |
| DAYSPM25 | Annual count of high PM2.5 days | 1,022 | 230 | 111.00 | 121.96 | 0.00 | 366.00 |
| BADDAYS | Annual count of high AQI days | 1,022 | 43 | 4.93 | 8.82 | 0.00 | 132.00 |

#### Table D2. Washington State Dataset Codebook & Summary Statistics

| Variable | Label | Obs | Unique | Mean | Std. dev. | Min | Max |
| --- | --- | --- | --- | --- | --- | --- | --- |
| EVENT_ID | Vote event identifier | 5,824 | 39 | 48.07 | 26.45 | 1 | 81 |
| MONTH | Vote month | 5,824 | 4 |  |  |  |  |
| YEAR | Year in which the vote event occurred | 5,824 | 13 | 2014.28 | 3.86 | 2010 | 2022 |
| ELECTIONDATE | Vote event date | 5,824 | 26 |  |  |  |  |
| FEBRUARY | 1=February vote event, 0=otherwise | 5,824 | 2 | 0.27 | 0.44 | 0 | 1 |
| APRIL | 1=April vote event, 0=otherwise | 5,824 | 2 | 0.14 | 0.35 | 0 | 1 |
| AUGUST | 1=August vote event, 0=otherwise | 5,824 | 2 | 0.47 | 0.50 | 0 | 1 |
| GENERAL | 1=General election vote event, 0=otherwise | 5,824 | 2 | 0.12 | 0.32 | 0 | 1 |
| PRES_YEAR | 1=2012, 2016, or 2020, 0= otherwise | 5,824 | 2 | 0.17 | 0.37 | 0 | 1 |
| COUNTYNAME | Precinct county name | 5,824 | 18 |  |  |  |  |
| STCOFIPS | Precinct county 5-character FIPS code | 5,824 | 18 |  |  |  |  |
| FSCSKEY | Federal library system identifier | 5,824 | 19 |  |  |  |  |
| LIBKEY | Numeric library system identifier constructed from FSCSKey and FIPS code | 5,824 | 23 |  |  |  |  |
| LIBRARYSYSTEM | Library system being voted on | 5,824 | 19 |  |  |  |  |
| PRECINCTNAME | Precinct name | 5,824 | 4,763 |  |  |  |  |
| PRECINCTCODE | Precinct code as string | 5,822 | 4,211 |  |  |  |  |
| PRECINCTNUMBER | Precinct code as number | 5,824 | 4,133 |  |  |  |  |
| ST_CODE | 10-character state code beginning with 2 character county string code | 5,824 | 4,524 |  |  |  |  |
| PRECBAR | Precinct code with \| used in some files | 5,824 | 4,559 |  |  |  |  |
| COUNTYFIPS | County FIPS code as string | 5,824 | 18 |  |  |  |  |
| NUMCOUNTY | County FIPS code as numeric | 5,824 | 18 | 41 | 15 | 5 | 75 |
| TRACT | 11-character Census block group identifier (State, County, Tract codes) | 5,824 | 877 |  |  |  |  |
| GEO_ID | 12-character Census block group identifier (State, County, Tract, Group #) | 5,824 | 1,836 |  |  |  |  |
| BLOCKGROUP | Census block group number | 5,821 | 9 |  |  |  |  |
| VOTINGAREA | Full area canvassed in referendum (e.g. county or school district) | 5,628 | 15 |  |  |  |  |
| ELECTIONTOPIC | Election Topic | 5,824 | 2 |  |  |  |  |
| ELECTIONDETAILS | Election details | 5,824 | 27 |  |  |  |  |
| VOTESYES | Yes votes in precinct | 5,824 | 471 | 135.23 | 90.31 | 0 | 630 |
| VOTESNO | No votes in precinct | 5,824 | 425 | 96.64 | 80.21 | 0 | 629 |
| TOTALVOTES | Total votes cast in library referendum | 5,824 | 745 | 231.87 | 156.73 | 0 | 1,228 |
| YESPERCENT | %YesVotes | 5,813 | 4,321 | 0.59 | 0.14 | 0 | 1 |
| DUMYES | 1= 50% or more yes votes, 0 otherwise | 5,824 | 2 | 0.73 | 0.45 | 0 | 1 |
| ODDSYES | Odds ratio calculated as %Yes / %No votes | 5,812 | 4,320 | 1.85 | 1.47 | 0 | 29 |
| LGODDS | Log transformation of ODDSYES | 5,809 | 4,319 | 0.40 | 0.63 | -2.40 | 3.37 |
| AMOUNT | Taxation amount as described on the ballot | 5,773 | 18 |  |  |  |  |
| LEVYTOTAL | Levy Total | 3,523 | 9 | 0.49 | 0.02 | 0.25 | 0.51 |
| LEVYPRIOR | Levy Prior | 323 | 5 | 0.35 | 0.02 | 0.25 | 0.39 |
| LEVYINCREASE | Levy Increase | 5,824 | 15 | 0.32 | 0.19 | 0.00 | 0.51 |
| LEVTAXTOTAL | Tax incidence from LEVYTOTAL | 3,416 | 984 | 176.18 | 74.24 | 5.00 | 546.85 |
| LEVTAXPRIOR | Tax incidence from LEVYTPRIOR | 317 | 110 | 95.31 | 42.44 | 38.63 | 282.77 |
| LEVTAXINCREASE | Tax incidence from LEVYTINCREASE | 5,628 | 1,597 | 129.23 | 83.17 | 0.00 | 546.85 |
| LNTAX | Log transformation of LEVYINCREASE | 5,615 | 1,596 | 4.61 | 0.81 | 0.99 | 6.30 |
| TAX_INCID | Tax incidence from LEVYINCREASE with missing imputed from LEVYTOTAL | 5,714 | 1,711 | 130.98 | 85.56 | 0.00 | 570.20 |
| TERM | Term levy is enacted if for limited term | 2,206 | 2 | 6.82 | 0.71 | 4.00 | 7.00 |
| REPUB_PE20 | Portion of precinct voting for republican presidential candidate in 2020 | 5,583 | 4,002 | 0.30 | 0.20 | 0.00 | 0.94 |
| REPUB_PE16 | Portion of precinct voting for republican presidential candidate in 2016 | 5,568 | 3,981 | 0.30 | 0.20 | 0.00 | 0.97 |
| REPUB_PE12 | Portion of precinct voting for republican presidential candidate in 2012 | 5,504 | 3,896 | 0.33 | 0.18 | 0.00 | 0.84 |
| AVG_REPUBLICAN | Average portion of precinct voting for republican presidential candidate across 2012-2020, multiplied by 100 | 5,629 | 4,286 | 31.40 | 19.01 | 0.00 | 91.11 |
| LIBINPRECINCT | 1=Library outlet in the voting precinct, 0 otherwise | 5,824 | 2 | 0.04 | 0.20 | 0.00 | 1.00 |
| DIST_MILES | Distance in miles from nearest edge of precinct to nearest library outlet | 5,824 | 5,536 | 1.14 | 1.34 | 0.00 | 16.73 |
| NEAR_FSCSKEY | Nearest outlet library system federal identifier | 5,824 | 35 |  |  |  |  |
| NEAR_FSCS_SEQ | Nearest outlet library branch federal identifier | 5,824 | 50 |  |  |  |  |
| NEAR_LIBID | Nearest outlet library branch state identifier | 5,824 | 217 |  |  |  |  |
| NEAR_LIBNAME | Nearest outlet library branch name | 5,824 | 218 |  |  |  |  |
| NEAR_ZIP | Nearest outlet location zip code | 5,824 | 193 | 98320 | 382 | 98002 | 99371 |
| NEAR_CNTY | Nearest outlet location county | 5,824 | 25 |  |  |  |  |
| NEAR_CNTYPOP | Nearest outlet county population | 5,824 | 52 | 1,410,608 | 789,831 | 10,939 | 2,252,782 |
| NEAR_LOCALE | Nearest outlet rural-urban locale code | 5,824 | 12 | 18.59 | 8.67 | 11 | 43 |
| NEAR_CENTRACT | Near outlet location Census tract | 5,824 | 211 |  |  |  |  |
| NEAR_CENBLOCK | Near outlet location Census block | 5,824 | 148 |  |  |  |  |
| NEAR_SQ_FEET | Nearest outlet square footage | 5,806 | 189 | 18,377 | 43,374 | 100 | 362,987 |
| NEAR_HOURS | Nearest outlet annual open hours | 5,795 | 158 | 2,713 | 690 | 72 | 3,848 |
| NEAR_WKS_OPEN | Nearest outlet annual weeks open | 5,807 | 13 | 51.11 | 5.01 | 4 | 52 |
| VOTENEAR_MISMATCH | 1=nearest outlet is not a branch of the library system being voted on, 0 otherwise | 5,824 | 2 | 0.06 | 0.24 | 0 | 1 |
| POPULATION | Count of Census bounded population | 5,705 | 1,449 | 3,428 | 2,120 | 0 | 9,870 |
| FPOPULATION | Flag tract-bounded value for population, 0=block group bounded | 5,705 | 2 | 0.50 | 0.50 | 0 | 1 |
| POPULATION_M | Count of Census bounded population, margin of error | 5,705 | 520 | 367.48 | 154.68 | 12 | 2324 |
| FPOPULATION_M | Flag tract-bounded value for population_m, 0=block group bounded | 5,705 | 2 | 0.50 | 0.50 | 0 | 1 |
| HOUSEHOLDS_CNT | Count of households | 5,705 | 1,158 | 1,525 | 1,016 | 0 | 6,719 |
| FHOUEHOLDS_CNT | Flag tract-bounded value for houeholds_cnt, 0=block group bounded | 5,705 | 2 | 0.50 | 0.50 | 0 | 1 |
| HOUSEHOLDS_CNTM | Count of households, margin of error | 5,705 | 222 | 96.15 | 48.21 | 12 | 452 |
| FHOUEHOLDS_CNTM | Flag tract-bounded value for houeholds_cntm, 0=block group bounded | 5,705 | 2 | 0.50 | 0.50 | 0 | 1 |
| MEDHOUSEVALUE | Median house value ($) estimate | 5,682 | 1,570 | $437,146 | $219,120 | $9,999 | $2,000,001 |
| FMEDHOUSEVALUE | Flag tract-bounded value for medhousevalue, 0=block group bounded | 5,705 | 2 | 0.52 | 0.50 | 0 | 1 |
| MEDHOUSEVALUE_M | Median house value ($) margin of error | 5,671 | 1,793 | $53,303 | $60,347 | $4,507 | $1,142,066 |
| FMEDHOUSEVALUE_M | Flag tract-bounded value for medhousevalue_m, 0=block group bounded | 5,705 | 2 | 0.52 | 0.50 | 0 | 1 |
| MEDHHINCOME | Median household income ($), estimate | 5,692 | 1,748 | $ 81,954 | $33,851 | $7,700 | $ 250,001 |
| FMEDHHINCOME | Flag tract-bounded value for medhhincome, 0=block group bounded | 5,705 | 2 | 0.51 | 0.50 | 0 | 1 |
| MEDHHINCOME_M | Median household income ($), margin of error | 5,692 | 1,764 | $17,121 | $14,514 | $909 | $120,425 |
| FMEDHHINCOME_M | Flag tract-bounded value for medhhincome_m, 0=block group bounded | 5,705 | 2 | 0.51 | 0.50 | 0 | 1 |
| MHHI_SCALED | Scaled median household income calculated MEDHHINCOME / 10000 | 5,692 | 1,748 | 8.20 | 3.39 | 0.77 | 25 |
| OWNOCC_CNT | Count of owner occupied housing units, estimate | 5,705 | 1,384 | 2,271 | 1,637 | 0 | 8,040 |
| FOWNOCC_CNT | Flag tract-bounded value for ownocc_cnt, 0=block group bounded | 5,705 | 2 | 0.50 | 0.50 | 0 | 1 |
| OWNOCC_CNTM | Count of owner occupied housing units, margin of error | 5,705 | 506 | 329.59 | 134.64 | 12 | 823 |
| FOWNOCC_CNTM | Flag tract-bounded value for ownocc_cntm, 0=block group bounded | 5,705 | 2 | 0.50 | 0.50 | 0 | 1 |
| OWNEROCC_PERC | % household owner occupied | 5,691 | 1,807 | 66.33 | 22.68 | 0 |  |
| OCC_CNT | Count of occupied households | 5,705 | 1,108 | 1,420 | 937 | 0 | 4,874 |
| FOCC_CNT | Flag tract-bounded value for occ_cnt, 0=block group bounded | 5,705 | 2 | 0.50 | 0.50 | 0 | 1 |
| OCC_CNTM | Count of occupied households, margin of error | 5,705 | 217 | 114.14 | 44.58 | 11 | 376 |
| FOCC_CNTM | Flag tract-bounded value for occ_cntm, 0=block group bounded | 5,705 | 2 | 0.50 | 0.50 | 0 | 1 |
| MALE_CNT | Count of male residents | 5,705 | 1,222 | 1,706 | 1,055 | 0 | 5,102 |
| FMALE_CNT | Flag tract-bounded value for male_cnt, 0=block group bounded | 5,705 | 2 | 0.50 | 0.50 | 0 | 1 |
| MALE_CNTM | Count of male residents, margin of error | 5,705 | 379 | 239.11 | 101.01 | 12 | 1,735 |
| FMALE_CNTM | Flag tract-bounded value for male_cntm, 0=block group bounded | 5,705 | 2 | 0.50 | 0.50 | 0 | 1 |
| MALE_PERC | % population were male | 5,700 | 1,809 | 49.88 | 4.85 | 22.53 | 100 |
| FEM85_CNT | Count of female residents 85 years or older | 5,705 | 172 | 38.74 | 57.59 | 0 | 952 |
| FFEM85_CNT | Flag tract-bounded value for fem85_cnt, 0=block group bounded | 5,705 | 2 | 0.50 | 0.50 | 0 | 1 |
| FEM85_CNTM | Count of female residents 85 years or older, margin of error | 5,705 | 119 | 38.32 | 45.90 | 2 | 1,206 |
| FFEM85_CNTM | Flag tract-bounded value for fem85_cntm, 0=block group bounded | 5,705 | 2 | 0.50 | 0.50 | 0 | 1 |
| FEM85_PERC | % population were female 85 or more years old | 5,700 | 1,274 | 1.20 | 1.65 | 0 | 21.30 |
| POVERTY_CNT | Count of household with poverty level incomes | 5,705 | 699 | 492.81 | 403.28 | 0 | 3,743 |
| FPOVERTY_CNT | Flag tract-bounded value for poverty_cnt, 0=block group bounded | 5,705 | 1 | 1.00 | 0.00 | 1 | 1 |
| POVERTY_CNTM | Count of household with poverty level incomes, margin of error | 5,705 | 440 | 226.14 | 145.28 | 8 | 1,972 |
| FPOVERTY_C~M | Flag tract-bounded value for poverty_cntm, 0=block group bounded | 5,705 | 1 | 1.00 | 0.00 | 1 | 1 |
| POVERTY_PERC | Poverty rate | 5,691 | 1,816 | 20.97 | 21.63 | 0 | 100 |
| GRAPI_PERC | Median gross rent as a percentage of household income, estimate | 5,678 | 330 | 29.10 | 7.67 | 9 | 51 |
| FGRAPI_PERC | Flag tract-bounded value for grapi_perc, 0=block group bounded | 5,705 | 2 | 0.56 | 0.50 | 0 | 1 |
| GRAPI_PERCM | Median gross rent as a percentage of household income, margin of error | 5,584 | 347 | 10.04 | 9.64 | 0.6 | 79.7 |
| FGRAPI_PERCM | Flag tract-bounded value for grapi_percm, 0=block group bounded | 5,705 | 2 | 0.59 | 0.49 | 0 | 1 |
| SMOCAPI_PERC | Median selected owner costs as a percentage of household income, estimate | 5,687 | 228 | 21.91 | 4.18 | 9 | 51 |
| FSMOCAPI_PERC | Flag tract-bounded value for smocapi_perc, 0=block group bounded | 5,705 | 2 | 0.51 | 0.50 | 0 | 1 |
| SMOCAPI_PERCM | Median selected owner costs as a percentage of household income, margin of error | 5,684 | 190 | 4.54 | 4.56 | 1.1 | 66.9 |
| FSMOCAPI_PERCM | Flag tract-bounded value for smocapi_percm, 0=block group bounded | 5,705 | 2 | 0.51 | 0.50 | 0 | 1 |
| UNEMPLOYED_PERC | Unemployment rate estimate | 5,700 | 1,384 | 4.12 | 3.22 | 0 | 28.2 |
| FUNEMPLOYED_PERC | Flag tract-bounded value for UNEMPLOYED_PERC, 0=block group bounded | 5,705 | 2 | 0.50 | 0.50 | 0 | 1 |
| BAPLUSPE | % population over 24 years with bachelor's degree or more | 5,700 | 1,702 | 44.05 | 21.66 | 0 | 94.98 |
| FBAPLUSPE | Flag tract-bounded value for BAPLUSPE, 0=block group bounded | 5,705 | 2 | 0.50 | 0.50 | 0 | 1 |
| FORBORNPE | % population born outside US States and US territories | 5,700 | 972 | 14.17 | 10.00 | 0 | 65.22 |
| FFORBORNPE | Flag tract-bounded value for FORBORNPE, 0=block group bounded | 5,705 | 1 | 1.00 | 0.00 | 1 | 1 |
| INDEX100 | Annual total days heat index 100 F degrees + | 5,824 | 2 | 0.00 | 0.05 | 0 | 4 |
| INDEX105 | Annual total days heat index 105 F degrees + | 5,824 | 2 | 0.00 | 0.04 | 0 | 3 |
| INDEX90 | Annual total days heat index 90 F degrees + | 5,824 | 18 | 3.08 | 3.59 | 0 | 25 |
| INDEX95 | Annual total days heat index 95 F degrees + | 5,824 | 6 | 0.31 | 0.69 | 0 | 8 |
| TEMP100 | Annual total days abs. temp 100 F degrees + | 5,824 | 4 | 0.04 | 0.30 | 0 | 5 |
| TEMP105 | Annual total days abs. temp 105 F degrees + | 5,824 | 1 | 0.00 | 0.00 | 0 | 0 |
| TEMP90 | Annual total days abs. temp 90 F degrees + | 5,824 | 30 | 2.39 | 6.84 | 0 | 41 |
| TEMP95 | Annual total days abs. temp 95 F degrees + | 5,824 | 12 | 0.47 | 1.94 | 0 | 24 |
| FHEAT | Flag county-bounded value for heat variables, 0=block group bounded | 5,824 | 2 | 0.31 | 0.46 | 0 | 1 |
| UNHEALTHYPM25 | Count of days with PM 2.5 levels at "unhealthy for sensitive groups days" | 5,713 | 7 | 2.68 | 2.65 | 0 | 10 |
| STCODE_NUM | Numeric version of ST_CODE, replacing string county prefix with numeric FIPS code | 5824 | 4,524 | 1500000000 | 4070000000 | 33000000 | 7500000000 |
| LIBNAME | Referendum library system name | 5824 | 19 | . | . | . | . |
| C_LEGBAS | Legal basis or governance structure of system: municipal, county, non-profit agency | 5824 | 2 | . | . | . | . |
| C_FSCS | Federal definition of a library met: Y for yes, N for no | 5824 | 2 | . | . | . | . |
| POPU_LSA | Population served by library | 5824 | 35 | 732,930 | 393,650 | 1,995 | 1,330,955 |
| POPU_UND | Population served by library less populations in overlapping service areas | 5824 | 35 | 732,930 | 393,650 | 1,995 | 1,330,955 |
| CENTLIB | Count of central libraries within library system | 5824 | 2 | 1 | 0 | 0 | 1 |
| BRANLIB | Count of branch libraries within library system | 5824 | 13 | 26 | 13 | 0 | 46 |
| BKMOB | Count of bookmobiles operated by library system | 5824 | 5 | 4 | 3 | 0 | 8 |
| MASTER | Count of master's degree holding librarians (FTE) | 5824 | 20 | 106 | 68 | 0 | 200 |
| LIBRARIA | Count of staff with "librarian" title (FTE) | 5824 | 24 | 107 | 67 | 0 | 200 |
| OTHPAID | Count of other paid staff (FTE) | 5824 | 26 | 347 | 180 | 0 | 566 |
| TOTSTAFF | Total paid staff (FTE) | 5824 | 31 | 454 | 245 | 0 | 766 |
| LOCGVT | Non-capital funding ($) from local government sources | 5824 | 37 | $50,900,000 | $30,000,000 | $ - | $ 89,300,000 |
| STGVT | Non-capital funding ($) from state government sources | 5824 | 5 | $ 20,053 | $35,570 | $ - | $ 83,229 |
| FEDGVT | Non-capital funding ($) from federal government sources | 5824 | 14 | $204,147 | $ 303,253 | $ - | $ 665,462 |
| OTHINCM | Non-capital funding ($) from non-government sources | 5824 | 36 | $3,046,692 | $3,327,513 | $ - | $ 10,200,000 |
| TOTINCM | Total non-capital revenues ($) | 5824 | 37 | $4,200,000 | $32,000,000 | $ 27,145 | $92,500,000 |
| SALARIES | Total salary or wage expenditures for referendum system ($) | 5804 | 24 | $6,600,000 | $14,500,000 | $130,878 | $42,700,000 |
| BENEFIT | Total employee benefit expenditures ($) | 5804 | 24 | $9,945,685 | $5,799,294 | $ 55,839 | $18,600,000 |
| STAFFEXP | Total staff (wage plus benefit) expenditures ($) | 5804 | 24 | $6,600,000 | $19,900,000 | $186,717 | $57,000,000 |
| PRMATEXP | Print material expenditures ($) | 5824 | 36 | $3,279,395 | $2,539,472 | $ 117 | $ 7,352,277 |
| ELMATEXP | Digital material expenditures ($) | 5824 | 26 | $2,585,021 | $ 1,767,549 | $ - | $ 4,533,859 |
| OTHMATEX | Other, A/V material expenditures ($) | 5824 | 28 | $ 547,197 | $ 479,874 | $ - | $ 1,266,986 |
| TOTEXPCO | Total collection expenditures ($) | 5824 | 36 | $6,411,613 | $4,114,645 | $ 117 | $ 11,900,000 |
| TOTOPEXP | Total operating expenditures ($) | 5824 | 37 | $ 54,000,000 | $32,000,000 | $ 23,977 | $ 92,500,000 |
| LCAP_REV | Capital funding ($) from local government | 5824 | 4 | $1,058,769 | $1,922,076 | $ - | $ 5,223,998 |
| SCAP_REV | Capital funding ($) from state government | 5824 | 1 | $ - | $ - | $ - | $ - |
| FCAP_REV | #N/A | 5824 | 1 | $ - | $ - | $ - | $ - |
| OCAP_REV | Capital funding ($) from non-government sources | 5824 | 7 | $ 142,851 | $ 205,785 | $ - | $ 1,799,898 |
| CAP_REV | Total capital funding ($) | 5824 | 8 | $1,201,620 | $2,106,724 | $ - | $ 5,753,221 |
| CAPITAL | Total capital expenditures ($) | 5824 | 26 | $5,705,671 | $7,441,389 | $ - | $ 18,500,000 |
| BKVOL | Total print books in collection | 5824 | 37 | 1,528,933 | 1,043,029 | 15,766 | 3,116,322 |
| EBOOK | Total ebooks in collection | 5824 | 32 | 136,136 | 144,988 | 0 | 424,013 |
| AUDIO_PH | Total physical audio items in collection | 5824 | 33 | 147,467 | 124,125 | 100 | 355,192 |
| AUDIO_DL | Total digital audio items in collection | 5824 | 31 | 40,400 | 37,142 | 0 | 103,971 |
| VIDEO_PH | Total physical video items in collection | 5824 | 35 | 91,777 | 62,856 | 550 | 183,472 |
| VIDEO_DL | Total digital video items in collection | 5824 | 13 | 20,038 | 39,943 | 0 | 107,984 |
| HRS_OPEN | Open hours summed across all library outlets annually | 5820 | 30 | 73,949 | 47,660 | 880 | 151,632 |
| VISITS | Visits to libraries summed across all library outlets annually | 5824 | 37 | 5,126,607 | 3,401,386 | 800 | 10,200,000 |
| REFERENC | Reference transactions summed across all library outlets annually | 5824 | 36 | 526,875 | 360,267 | 7 | 984,731 |
| REGBOR | Count of library card holders | 5824 | 37 | 513,612 | 334,756 | 800 | 1,041,515 |
| TOTCIR | Total items borrowed digitally and physically, summed across all library outlets annually | 5824 | 37 | 11,100,000 | 7,231,998 | 7,415 | 22,400,000 |
| KIDCIRCL | Total children's items borrowed summed across all library outlets annually | 5824 | 37 | 2,931,294 | 1,695,317 | 1,280 | 5,376,237 |
| TOTCOLL | Total of all physical items held within library systems across all outlets | 5824 | 37 | 1,768,175 | 1,161,294 | 13,390 | 3,503,972 |
| LOANTO | Total physical items lent to a library in a different library system, annually | 5824 | 31 | 8,053 | 5,403 | 0 | 275,402 |
| LOANFM | Total physical items borrowed to a library in a different library system, annually | 5824 | 34 | 8,940 | 8,115 | 45 | 116,483 |
| TOTPRO | Total program instances hosted summed across all library outlets annually | 5821 | 33 | 8,604 | 4,498 | 4 | 15,051 |
| YAPRO | Total teen or young adult program instances hosted summed across all library outlets annually | 5726 | 23 | 786 | 578 | 0 | 1,707 |
| TOTATTEN | Total program attendees summed across all library outlets annually | 5821 | 35 | 229,981 | 136,486 | 21 | 429,483 |
| YAATTEN | Total teen or young adult program attendees summed across all library outlets annually | 5726 | 22 | 14,858 | 9,380 | 0 | 26,177 |
| GPTERMS | Total public access computer stations summed across all library outlets annually | 5821 | 26 | 680 | 382 | 5 | 1,100 |
| PITUSR | Total uses of public access computer stations summed across all library outlets annually | 5824 | 37 | 1,406,593 | 1,444,744 | 1,057 | 3,880,000 |
| WIFISESS | Total times wireless internet was accessed via a personal device, summed across all library outlets annually | 2526 | 17 | 901,364 | 768,449 | 0 | 1,793,762 |
| KIDPRO | Total children's program instances hosted summed across all library outlets annually | 5821 | 33 | 5,653 | 3,995 | 4 | 12,257 |
| KIDATTEN | Total children's program attendees summed across all library outlets annually | 5821 | 35 | 177,181 | 125,915 | 21 | 385,493 |
| LOCALE | Locale code assigned to library administration location: 11=urban, 21=suburban, 31=small town, 41=rural | 5824 | 11 | 19 | 8.61 | 11 | 43 |
| AVG_SQFT | Average square footage within library system | 5824 | 22 | 15,588 | 7,093 | 1,591 | 57,757 |
| TOT_SQFT | Total square footage of all outlets within library system | 5824 | 22 | 407,408 | 227,770 | 3,487 | 612,183 |
| SINGMULT | 1= single outlet library system; 2 = multiple outlet library system | 5824 | 2 | 2 | 0.10 | 1 | 2 |
| STFHRS | Total annual staff hours summed across all outlets calculated as: TOTSTAF * 2080 | 5824 | 31 | 943,797 | 510,016 | 0 | 1,593,426 |
| DENSITY | Based on locale code: 1=Urban, 2=Suburban, 3=Small Town, 4=Rural | 5824 | 4 | 2 | 0.84 | 1 | 4 |
| LOCFUNDPERCAP | Local funding per capita calculated: LOCGVT / POP_LSA | 5824 | 37 | $ 65 | $ 20 | $ - | $ 173 |
| COMPUSE | Total uses of library provided internet, either via computing station or wi-fi connection, calculated: pitusr + wifisess | 5824 | 37 | 1,797,535 | 1,401,031 | 1,057 | 3,880,000 |
| REGCAPITA | Library card holders per capita calculated: REGBOR / POP_LSA | 5824 | 37 | 1 | 0.16 | 0 | 2 |
| WAGESTAFF | Wages per FTE staff persons calculated: SALARIES/TOTSTAFF | 5804 | 24 | $77,434 | $ 13,382 | $47,049 | $ 97,161 |
| STAFCAPITA | FTE per 1,000 service population calculated: TOTSTAFF / POP_LSA | 5824 | 35 | 0.61 | 0.16 | 0.00 | 1.62 |
| TOTOUT | Count of total outlets in library system calculated: CENLIB + BRANLIB | 5824 | 13 | 26 | 13.22 | 1 | 46 |

## Appendix E. Data & Methods Detail

#### Tax Incidence Calculation

Taxation schemes are typically based on either the value of a real estate owner’s property or a purchaser’s consumption. Votes where the individual tax burden is assumed within a sales tax scheme were excluded from tax incidence calculation. Operational levies and millages tax incidence calculation was straight forward in many states, including Washington, where the ballot includes the specific household price per $1,000 of assessed property taxes. The calculation assumes 100% assessment of property value by county assessors and holds true, according to Secretary of State websites, in Louisiana and Washington. All tax incidence for voters within a library system or precinct *i* is a measure of millage or levy rate from ballot initiative *b*, multiplied by median house value within the vote count geography (mapped to *i*), scaled as indicated in the ballot. This is shown in its most common form:

$tax incidence_{i}=levy_{b}*(\frac{housevalue_{i}}{1,000})$	(1)

There are states (e.g., NY), with common vote language giving the total funding amount rather than the household price. A 2022 ballot in Cherry Valley (NY) offers a good example:

Pursuant to Education Law, Section 259, the Board of Education of the Cherry Valley-Springfield Central school District is authorized to levy and collect an annual tax, year after year, separate and apart from the annual school district budget, in the amount of $60,000…

(CV-S CS Board of Education, 2022)

For these vote events where only the total annual funding allocation is known, the allocation is scaled first by number of households in the library service area:

$tax incidence_{i}=\frac{{total allocation}_{b}}{{(service population}_{b}/{household size}_{i})}$	(2)

#### Empirical Strategy – Estimating probabilities with proportion data

Analysis proceeds with how socio-ecological characteristics, risks, and outcomes contribute to the probability that a library tax increase wins at the ballot box using a log-odds model. Empirical research into the utility of passing a public good referendum flows from a framework established by Deacon & Shapiro (1975) and others (e.g., Banzhaf et al., 2010; Liu, 2022). In that work, the dependent variable is theoretically framed as a discrete moment of choice: yes or no to an expense. While based in that same choice moment, the structure of our data is the proportion of positive discrete decisions within an aggregated space-time boundary. Recent work on implementing estimation with fractional or proportion data consistent with the data generating process (discrete choices) has shown that intentionally selecting a distribution and applying a logit link function gives the theoretically correct and least biased results (Villadsen & Wulff, 2021).

In the only extant prior studies on vote determinants for libraries, and consistent with the bulk of referendum studies, the dependent variable (portion of yes votes) is recalculated as a probability ratio and log transformed, referred to as the log odds ratio, and given as $y_{it}$ below:

$y_{it}\equiv\ln(p_{it}/(1-p_{it})$	(3)

Where $p_{it}$ is the portion of yes votes in library district or precinct *i* in time *t*. There are voting precincts in the Washington data with 0% and 100% yes votes (values equal to 0 and 1) that become missing when transformed. While estimation using the transformed dependent variable is included in Appendix F as a demonstration of consistency and normality in the data, this standard is the not the primary model employed here.

Rather than transforming the dependent variable, the proportional data is retained as is. The primary model assumes a Bernoulli data generating process (distributed within the binomial family) and applies a logit link function to the expectation of yes votes. This follows Papke and Woolridge (1996) for a fractional linear regression approach to estimating expectations of proportional data bounded by 0 and 1.

$logit\left\{ E(y) \right\}=\boldsymbol{x\beta}$	(4)

Where the distribution of y is not assumed to be normal and the estimation no longer holds the strict independence assumptions required by OLS. Instead, estimation follows a general linear model by maximum likelihood with assumed binomial distribution. The link function with expectation of y given as *μ*, defined as:

$\eta=ln\{\frac{\mu}{1-\mu}\}$	(5)

This is identical to $y_{it}$ in equation (3), and where data are distributed normally, one could expect the models to produce near identical results within varying confidence intervals.

As discussed in section “Library Funding Referendum Context”, geographic population and voting population differ. In estimating the determinants of voter behavior, the model is specifically concerned with individuals who vote in library referendum events. Observations are weighted ($w_{i}$) by the population of voters ($M$) (TOTALVOTES) defined as voters in precinct i divided by the sum of all voters across precincts i through k:

$w_{i}=Mv_{i}/\left( \sum_{k} v_{k} \right)$	(6)

Following Burkhardt & Chan (2017), the log likelihood function takes the following form:

$L=\sum_{i=1}^{I} w_{i}\left[ p_{it}\log\left( \boldsymbol{x\beta} \right)\boldsymbol{+}\left( 1-p_{it} \right)log(1-\boldsymbol{x\beta)} \right]$	(7)

The primary estimation specification for both datasets analyzed here is then:

$$p_{it}=\alpha^{'}TaxIncidence_{it}+\beta^{'}Household_{it}+\gamma^{'}Environment_{it}+\kappa^{'}Partisanship_{i}+$$

$\delta^{'}Spatial_{it}+\theta_{i}+\varepsilon_{j}$	(8)

Where:

$p_{it}$: portion of yes votes within the voting boundary and event time (full event in national data and precinct in Washington data), bounded by 0 and 1.

$TaxIncidence_{it}$: property tax cost associated with voting yes calculated for the geography where and when the vote occurred.

$Household_{it}$: vector of household member characteristics aggregated as proportions within the geography where and when the vote occurred (e.g. percent residents with Bachelor’s degree or more in educational attainment, BAPLUSPE).

$Environment_{it}$: vector of environmental risks and burdens as either counts or indexes associated with voting place and time.

$Partisanship_{i}$: Republican vote share for a geography averaged over presidential election events in 2012, 2016, and 2020.

$Spatial_{i}$:vector of spatial characteristics related to voting precinct and its nearest library building. This is only specified in the Washington analysis.

$\theta_{i}$: vector of time invariant fixed effects for the institutional arrangement and context of the library system being voted on.

The errors are robust and clustered at the library system, *j*, level and the model is weighted by the number of voters participating in the referendum.

#### Empirical Strategy – Estimating Willingness to Pay with precinct-level proportion data

There are a few library systems in Washington that cover enough precincts where they have enough variation themselves so that household willingness to pay (WTP) values could be estimated when the precinct results were pooled over the study years. Again, the empirical model used is the log-odds regression on precinct level specifications named above. Following Appendix H.1 in Anderson et al., (2023), the estimated coefficient $\alpha$ on tax incidence represents the marginal utility of income that is used to scale the log-odds of voting yes within a precinct, resulting in a precinct-level mean annual household WTP estimate of:

$\bar{WTP_{i}}=y_{i}/\hat{\alpha_{j}}$	(9)

To summarize, precinct-level voting data requires a novel WTP estimation approach.

By way of comparison, in the most common WTP estimation approach (e.g., Cuccia, 2020) using survey-based hypothetical referendum data, a sample of people, each with varying incomes are each making a choice given a random (within pre-selected bounds) annual taxation amount. These survey data are then *variation rich* at the income-tax incidence level. In a real referendum setting, for the 250 voters in precinct *i*, voter *a* may have an income of $100k facing a tax incidence of $100 while voter *b* may have an income of $200k but in a smaller home with a tax incidence of $80. Both voters in precinct *i* in which 59% of voters said yes, are aggregated into block group matched to precinct *i* with median income of $90k, house value of $400k, and property value-based tax incidence of $131. The structure of real precinct level data can be imagined as survey results that have been collapsed by income-tax incidence level, representing a few or many hundreds of responses to a consequential payment question. Following Anderson, et al. (2023) the assumption is that this aggregated data is sufficiently modeled by equation 9.

Following Anderson, et al. (2022), precinct level WTP is calculated as the log odds divided by the marginal utility of income, as in equation (9):

$\bar{WTP_{i}}=y_{i}/\hat{\alpha_{j}}$	(10)

With $y_{i}$ defined as the transformation of proportion of yes votes recalculated as a probability ratio and log transformed (the log odds ratio), as described in equation (3) but pooled over time,

$y_{i}=\ln(p_{i}/(1-p_{i})$	(11)

Where $p_{i}$ is the portion of yes votes in district or precinct *i*. For precinct mean WTP, the marginal utility of income is measured by the coefficient on *TAX_INCID*, $\hat{\alpha}_{j}$, estimated with weights for total votes within precinct *i* and restricted to the sample of precincts voting for system *j*. Results are given for $\hat{\alpha}_{j}$ with significance as low as 90%.

Here, to decompose WTP for a specific library system into the loss of value for every mile traveled to get to a library outlet the coefficient β on *DIST_MILES* specified in Model 6 is divided by $\hat{\alpha}$:

$\frac{\beta^{'}DIST\_MILES}{\alpha'TAX\_INCID}$	(12)

Table F5 shows the results of this estimation with a more complete discussion of the results, to supplement what can be found in the main text.

## Appendix F. Additional Results

#### Table F1. Regression Approach Comparison, National Data: OLS, Tobit, MLE with Logit Link

|  | OLS  (~Normal) | Censored Tobit  (~Normal) | Logit Link  (~Bernoulli) |
| --- | --- | --- | --- |
| Dependent | LGODDS | LGODDS | YES_PERC |
| TAXINCID_MIXED | -0.0015*** | -0.0011** | -0.0014*** |
|  | (-4.07) | (-3.02) | (-3.96) |
| AVGREP | 0.0000 | 0.0000 | 0.0000 |
|  | (-1.49) | (-1.61) | (-1.46) |
| BAPLUSPE | 0.0091*** | 0.0082*** | 0.0087*** |
|  | (-5.16) | (-4.81) | (-5.31) |
| STABLEPE | -0.0057** | -0.0066** | -0.0055** |
|  | (-2.73) | (-2.98) | (-2.78) |
| FORBORNPE | -0.0052 | -0.0001 | -0.0043 |
|  | (-1.78) | (-0.02) | (-1.48) |
| ELDHHPE | 0.0061 | 0.0061 | 0.0056 |
|  | (-1.79) | (-1.72) | (-1.72) |
| CRF_VALUE | 0.2200 | 0.1990 | 0.2220 |
|  | (-0.97) | -0.89 | -1.04 |
| INDEX90 | 0.0053** | 0.0040* | 0.0049** |
|  | (-3.29) | -2.11 | -2.89 |
| _cons | 0.7790 | 0.7530 | 0.7330 |
|  | (-1.91) | -1.8 | -1.87 |
| var(e.lgod~) | 0.0742*** |  |  |
|  | (-6.08) |  |  |
| N | 977 | 977 | 982 |
| t statistics in parentheses | |  |  |
| * p<0.05, ** p<0.01, *** p<0.001 | | |  |

Note. Model estimations are limited to observations with estimated tax incidence of less than $300. There are 90 vote events with mean tax incidence calculated for annual sums above $300, but the author knows of very few places where such an expectation is feasible. All models have standard errors clustered at the library system level.

#### Table F2. Regression Approach Comparison, Washington Data: OLS, Tobit, MLE with Logit Link

|  | | OLS  (~Normal) | | | Censored Tobit  (~Normal) | | | Logit Link  (~Bernoulli) | | |  |
| --- | --- | --- | --- | --- | --- | --- | --- | --- | --- | --- | --- |
| Dependent | LGODDS | | | | LGODDS | | | YESPERCENT | | |  |
| TAX_INCID | | | -0.0018* | | | -0.0017* | | | -0.0017** | | |
|  | | | (-2.57) | | | (-2.46) | | | (-2.65) | | |
| AVG_REPUBLICAN | | | -0.0138*** | | | -0.0144*** | | | -0.0134*** | | |
|  | | | (-6.34) | | | (-5.39) | | | (-6.15) | | |
| LIBINPRECINCT | | | 0.127*** | | | 0.148*** | | | 0.131*** | | |
|  | | | (-4.26) | | | (-5.31) | | | (-4.76) | | |
| DIST_MILES | | | -0.0456*** | | | -0.0460*** | | | -0.0438*** | | |
|  | | | (-6.60) | | | (-5.07) | | | (-6.75) | | |
| VOTENEAR_MISMATCH | | | -0.124* | | | -0.108 | | | -0.124** | | |
|  | | | (-2.84) | | | (-1.86) | | | (-2.79) | | |
| UNHEALTHYPM25 | | | -0.0194 | | | -0.014 | | | -0.0197 | | |
|  | | | (-0.81) | | | (-0.58) | | | (-0.83) | | |
| INDEX95 | | | 0.0356 | | | 0.043 | | | 0.0361 | | |
|  | | | (-1.23) | | | (-1.62) | | | (-1.26) | | |
| BAPLUSPE | | | 0.0082** | | | 0.0087*** | | | 0.0082*** | | |
|  | | | (-3.94) | | | (-4.62) | | | (-4.04) | | |
| OWNEROCC_PERC | | | -0.0038*** | | | -0.0041*** | | | -0.0038*** | | |
|  | | | (-10.09) | | | (-13.76) | | | (-10.92) | | |
| SMOCAPI_PERC | | | -0.0076*** | | | -0.0091*** | | | -0.0078*** | | |
|  | | | (-4.91) | | | (-5.29) | | | (-4.52) | | |
| FORBORNPE | | | -0.0071** | | | -0.0064** | | | -0.0070** | | |
|  | | | (-3.24) | | | (-2.76) | | | (-3.09) | | |
| UNEMPLOYED_PERC | | | -0.0054* | | | -0.0033 | | | -0.0052* | | |
|  | | | (-2.18) | | | (-1.19) | | | (-2.03) | | |
| _cons | | | 1.251*** | | | 1.207*** | | | 1.223*** | | |
|  | | | (-8.63) | | | (-8.64) | | | (-8.45) | | |
| var(e.lgod~) | | | 0.101*** | | | | | | | | |
|  | | | (-4.68) | | | | | | | | |
| N | | | 5,136 | | | 5,136 | | | 5,137 | | |
| t statistics in parentheses | | | |  | | |  | | |  |  |
| * p<0.05, ** p<0.01, *** p<0.001 | | | | | | |  | | |  |  |

Note. Model estimations are limited to observations with estimated tax incidence of less than $300. There are 385 precinct-event observations with mean tax incidence calculated for annual sums above $300. All models have standard errors clustered at the library system level.

#### Table F3. Full Main Model Regression Results: National Data

| YES_PERC | Coefficient | | Robust St. Error | | Z | | P>Z | | [95% conf. interval] | | | |  |
| --- | --- | --- | --- | --- | --- | --- | --- | --- | --- | --- | --- | --- | --- |
| TAXINCID_MIXED | -0.00159 | | 0.0004 | | -4 | | 0 | | -0.00237 | | -0.00081 | |  |
| FUNDCAPITA | 0.00385 | | 0.0013 | | 2.96 | | 0.003 | | 0.0013 | | 0.006403 | |  |
| AVGREP | -1.3E-05 | | 2.5E-05 | | -0.52 | | 0.6 | | -6.3E-05 | | 3.62E-05 | |  |
| BAPLUSPE | 0.00364 | | 0.00152 | | 2.39 | | 0.017 | | 0.000655 | | 0.006631 | |  |
| STABLEPE | -0.00492 | | 0.00225 | | -2.18 | | 0.029 | | -0.00933 | | -0.0005 | |  |
| ELDHHPE | 0.0061 | | 0.0033 | | 1.85 | | 0.065 | | -0.00037 | | 0.012567 | |  |
| INDEX100 | 0.01798 | | 0.00377 | | 4.77 | | 0 | | 0.010588 | | 0.025371 | |  |
|  |  | |  | |  | |  | |  | |  | |  |
| SERVICE_GEO | | |  | |  | |  | |  | |  | |  |
| CITY | 0.15419 | | 0.22248 | | 0.69 | | 0.488 | | -0.28186 | | 0.590232 | |  |
| COUNTY | 0.12531 | | 0.2281 | | 0.55 | | 0.583 | | -0.32176 | | 0.572387 | |  |
| LIBRARY DISTRICT | 0.16695 | | 0.21248 | | 0.79 | | 0.432 | | -0.24951 | | 0.5834 | |  |
| MULTI-JURISDICTION | -0.68117 | | 0.27125 | | -2.51 | | 0.012 | | -1.21281 | | -0.14952 | |  |
| NON-PROFIT | 0.12704 | | 0.23048 | | 0.55 | | 0.581 | | -0.32468 | | 0.578774 | |  |
| SCHOOL DISTRICT | 0.30025 | | 0.23864 | | 1.26 | | 0.208 | | -0.16748 | | 0.767978 | |  |
| OTHER | -0.00328 | | 0.24219 | | -0.01 | | 0.989 | | -0.47797 | | 0.471406 | |  |
|  |  | |  | |  | |  | |  | |  | |  |
| LOCALE |  | |  | |  | |  | |  | |  | |  |
| 12 | -0.14016 | | 0.12295 | | -1.14 | | 0.254 | | -0.38113 | | 0.10082 | |  |
| 13 | -0.1838 | | 0.14657 | | -1.25 | | 0.21 | | -0.47107 | | 0.103461 | |  |
| 21 | -0.23879 | | 0.13076 | | -1.83 | | 0.068 | | -0.49507 | | 0.017495 | |  |
| 22 | -0.31204 | | 0.17622 | | -1.77 | | 0.077 | | -0.65743 | | 0.03334 | |  |
| 23 | -0.1272 | | 0.13522 | | -0.94 | | 0.347 | | -0.39223 | | 0.13782 | |  |
| 31 | -0.08275 | | 0.14424 | | -0.57 | | 0.566 | | -0.36546 | | 0.199948 | |  |
| 32 | -0.14749 | | 0.15315 | | -0.96 | | 0.336 | | -0.44765 | | 0.15267 | |  |
| 33 | 0.07459 | | 0.1532 | | 0.49 | | 0.626 | | -0.22567 | | 0.374843 | |  |
| 41 | -0.33718 | | 0.15463 | | -2.18 | | 0.029 | | -0.64025 | | -0.0341 | |  |
| 42 | -0.19593 | | 0.14829 | | -1.32 | | 0.186 | | -0.48658 | | 0.094718 | |  |
| 43 | -0.32101 | | 0.15853 | | -2.02 | | 0.043 | | -0.63173 | | -0.01029 | |  |
|  |  | |  | |  | |  | |  | |  | |  |
| 2.SINGMULT | 0.01269 | | 0.05411 | | 0.23 | | 0.815 | | -0.09336 | | 0.118736 | |  |
|  |  | |  | |  | |  | |  | |  | |  |
| NUMSTATE |  | |  | |  | |  | |  | |  | |  |
| 8 | -0.49047 | | 0.14679 | | -3.34 | | 0.001 | | -0.77816 | | -0.20277 | |  |
| 9 | -0.42 | | 0.20433 | | -2.06 | | 0.04 | | -0.82048 | | -0.01952 | |  |
| 16 | -0.54373 | | 0.23303 | | -2.33 | | 0.02 | | -1.00046 | | -0.087 | |  |
| 17 | -0.63371 | | 0.26858 | | -2.36 | | 0.018 | | -1.16013 | | -0.1073 | |  |
| 19 | -0.76254 | | 0.16914 | | -4.51 | | 0 | | -1.09406 | | -0.43102 | |  |
| 22 | -1.61468 | | 0.27687 | | -5.83 | | 0 | | -2.15734 | | -1.07202 | |  |
| 23 | -0.27241 | | 0.22648 | | -1.2 | | 0.229 | | -0.71631 | | 0.171484 | |  |
| 24 | 0.88608 | | 0.18722 | | 4.73 | | 0 | | 0.519144 | | 1.253025 | |  |
| 26 | 0.04295 | | 0.16893 | | 0.25 | | 0.799 | | -0.28814 | | 0.374041 | |  |
| 30 | 0.18681 | | 0.20828 | | 0.9 | | 0.37 | | -0.22141 | | 0.595028 | |  |
| 31 | -1.10334 | | 0.2116 | | -5.21 | | 0 | | -1.51807 | | -0.68862 | |  |
| 34 | -0.67618 | | 0.18619 | | -3.63 | | 0 | | -1.0411 | | -0.31125 | |  |
| 36 | -0.00506 | | 0.1826 | | -0.03 | | 0.978 | | -0.36294 | | 0.352829 | |  |
| 39 | 0.15175 | | 0.1821 | | 0.83 | | 0.405 | | -0.20515 | | 0.508654 | |  |
| 41 | -0.43234 | | 0.16528 | | -2.62 | | 0.009 | | -0.75628 | | -0.10839 | |  |
| 42 | 0.07644 | | 0.19892 | | 0.38 | | 0.701 | | -0.31343 | | 0.466319 | |  |
| 45 | -0.10655 | | 0.192 | | -0.55 | | 0.579 | | -0.48286 | | 0.269754 | |  |
| 48 | -1.16228 | | 0.21193 | | -5.48 | | 0 | | -1.57765 | | -0.74692 | |  |
| 49 | -0.84296 | | 0.23589 | | -3.57 | | 0 | | -1.30529 | | -0.38062 | |  |
| 53 | -0.31759 | | 0.16208 | | -1.96 | | 0.05 | | -0.63526 | | 7.63E-05 | |  |
| 54 | 0.09971 | | 0.23266 | | 0.43 | | 0.668 | | -0.3563 | | 0.55573 | |  |
|  |  | |  | |  | |  | |  | |  | |  |
| YEAR |  | |  | |  | |  | |  | |  | |  |
| 2011 | -0.41361 | | 0.1099 | | -3.76 | | 0 | | -0.62901 | | -0.19822 | |  |
| 2012 | -0.224 | | 0.09306 | | -2.41 | | 0.016 | | -0.4064 | | -0.04159 | |  |
| 2013 | -0.01924 | | 0.0817 | | -0.24 | | 0.814 | | -0.17937 | | 0.140883 | |  |
| 2014 | 0.04872 | | 0.06899 | | 0.71 | | 0.48 | | -0.08649 | | 0.183927 | |  |
| 2015 | 0.09434 | | 0.0688 | | 1.37 | | 0.17 | | -0.04051 | | 0.22918 | |  |
| 2016 | 0.12214 | | 0.06421 | | 1.9 | | 0.057 | | -0.00371 | | 0.247994 | |  |
| 2017 | 0.39327 | | 0.07321 | | 5.37 | | 0 | | 0.249791 | | 0.536755 | |  |
| 2018 | 0.07822 | | 0.049 | | 1.6 | | 0.11 | | -0.01781 | | 0.174245 | |  |
| 2019 | 0.1111 | | 0.10709 | | 1.04 | | 0.3 | | -0.09879 | | 0.32098 | |  |
| 2020 | 0.10328 | | 0.05299 | | 1.95 | | 0.051 | | -0.00058 | | 0.207143 | |  |
| 2021 | 0.11827 | | 0.10029 | | 1.18 | | 0.238 | | -0.0783 | | 0.314837 | |  |
|  |  | |  | |  | |  | |  | |  | |  |
| 1.GENERAL | -0.12414 | | 0.04603 | | -2.7 | | 0.007 | | -0.21435 | | -0.03393 | |  |
| _CONS | 0.85976 | | 0.27549 | | 3.12 | | 0.002 | | 0.319814 | | 1.399704 | |  |
|  | |  | |  | |  | |  | |  | |  | |

#### Table F4. Full Main Model Regression Results – Washington Data

|  | Coefficient | Robust std. err. | z | P>z | [95% conf. interval] | |
| --- | --- | --- | --- | --- | --- | --- |
| TAX_INCID | -0.002128 | 0.0005654 | -3.76 | 0 | -0.003236 | -0.00102 |
| LIBINPRECINCT | 0.1262759 | 0.0281615 | 4.48 | 0 | 0.0710802 | 0.1814715 |
| DIST_MILES | -0.03889 | 0.0074583 | -5.21 | 0 | -0.053508 | -0.024272 |
| 1.VOTENEAR_ MISMATCH | -0.137602 | 0.063558 | -2.16 | 0.03 | -0.262173 | -0.013031 |
| AVG_ REPUBLICAN | -0.014243 | 0.0024022 | -5.93 | 0 | -0.018951 | -0.009535 |
| BAPLUSPE | 0.010309 | 0.0015563 | 6.62 | 0 | 0.0072588 | 0.0133592 |
| OWNEROCC_ PERC | -0.003958 | 0.0005243 | -7.55 | 0 | -0.004985 | -0.00293 |
| SMOCAPI_PERC | -0.012536 | 0.0034379 | -3.65 | 0 | -0.019274 | -0.005798 |
| FORBORNPE | -0.006619 | 0.0031023 | -2.13 | 0.033 | -0.012699 | -0.000538 |
| UNEMPLOYED_ PERC | -0.014404 | 0.0058337 | -2.47 | 0.014 | -0.025838 | -0.00297 |
| UNHEALTHY  PM25 | -0.049401 | 0.0318526 | -1.55 | 0.121 | -0.111831 | 0.0130287 |
| INDEX90 | 0.0224061 | 0.0113549 | 1.97 | 0.048 | 0.0001508 | 0.0446614 |
|  |  |  |  |  |  |  |
| NEAR_LOCALE |  |  |  |  |  |  |
| 12 | 0.2968083 | 0.103507 | 2.87 | 0.004 | 0.0939382 | 0.4996784 |
| 13 | 0.2287169 | 0.1106274 | 2.07 | 0.039 | 0.0118912 | 0.4455426 |
| 21 | 0.2099417 | 0.1046592 | 2.01 | 0.045 | 0.0048134 | 0.41507 |
| 22 | 0.0718283 | 0.1512935 | 0.47 | 0.635 | -0.224702 | 0.3683582 |
| 23 | 0.2921444 | 0.2009649 | 1.45 | 0.146 | -0.10174 | 0.6860283 |
| 31 | 0.2967162 | 0.0941524 | 3.15 | 0.002 | 0.1121809 | 0.4812515 |
| 32 | 0.4410047 | 0.1610205 | 2.74 | 0.006 | 0.1254104 | 0.7565991 |
| 33 | 0.3670653 | 0.1694142 | 2.17 | 0.03 | 0.0350196 | 0.699111 |
| 41 | 0.2710912 | 0.1036278 | 2.62 | 0.009 | 0.0679845 | 0.474198 |
| 42 | 0.215322 | 0.1097216 | 1.96 | 0.05 | 0.0002715 | 0.4303724 |
| 43 | 0.3666805 | 0.1601156 | 2.29 | 0.022 | 0.0528597 | 0.6805013 |
|  |  |  |  |  |  |  |
| 1.GENERAL | 0.0019298 | 0.069704 | 0.03 | 0.978 | -0.134688 | 0.1385472 |
| _cons | 1.314114 | 0.1429229 | 9.19 | 0 | 1.03399 | 1.594237 |

#### Willingness to Pay (WTP) Results

Table F5, shows the calculation results for library systems where the precinct level vote shares varied enough that robust estimates could be converged to with a strongly restricted sample. Library system mean household WTP was calculated as both the direct calculation from equation (10) above, as the division of precinct log-odds by system-wide regression $\hat{\alpha}$, and as a mean weighted by the total number of votes cast within each precinct. As expected, the lower the total number of votes cast, the less variation between WTP estimations.

Point estimates of annual household WTP range from $25 to $65, expanding to $4 to $99 when incorporating the outer limits of the standard deviations. The decomposition of value for distance to the nearest library building is not weighted because it doesn’t incorporate precinct level information directly but instead is a ratio of maximized estimates from the overall general linear model. Table 3 includes decomposition results where the $\hat{\beta}$ is statistically significant if $\hat{\alpha}$ is estimated as a negative, following statewide results and economic theory. Even so, where there is no WTP calculated, the $\hat{\alpha}$ used in the calculation of distance cost has a confidence interval that crosses 0, and should be treated as illustrative rather than absolute.

#### Table F5. Willingness to Pay Results by Library System, pooled over years

| Library ID | Precincts | Votes | % Yes | α'TAX_INCID | β'DIST_ MILES | WTP | Weighted WTP | Distance Cost | |  |
| --- | --- | --- | --- | --- | --- | --- | --- | --- | --- | --- |
| 90053 | 80 | 24,194 | 60% | -0.011^+^ | -0.037^*^ | $ 35.31 | $ 33.00 | -$ 3.27 |  |  |
|  |  |  | 0.08 | 0.006 | 0.015 | 31.26 | 26.70 |  |  |  |
| 15003 | 16 | 6,045 | 61% | -0.018^***^ | 0.730^***^ | $ 24.87 | $ 24.94 | $ 40.23 | | |
|  |  |  | 0.05 | 0.002 | 0.138 | 11.08 | 10.41 |  | | |
| 350060 | 191 | 59,624 | 64% | -0.010^**^ | -0.043^**^ | $ 59.86 | $ 64.72 | -$ 4.23 | | |
|  |  |  | 0.09 | 0.003 | 0.013 | 43.32 | 42.66 |  |  |  |
| 390058 | 28 | 5,362 | 60% | -0.003 | -0.028* |  |  | -$ 10.71 |  |  |
|  |  |  | 0.10 | 0.003 | 0.011 |  |  |  |  |  |
| 530063 | 346 | 197,541 | 50% | -0.000 | -0.027* |  |  | -$ 77.75 |  |  |
|  |  |  | 0.07 | 0.000 | 0.013 |  |  |  |  |  |
| 610065 | 628 | 95,137 | 48% | -0.001 | -0.057*** |  |  | -$ 90.74 |  |  |
|  |  |  | 0.10 | 0.000 | 0.013 |  |  |  |  |  |
| 630066 | 352 | 114,180 | 55% | -0.001 | -0.044*** |  |  | -$ 77.29 |  |  |
|  |  |  | 0.08 | 0.000 | 0.009 |  |  |  |  |  |

*Sources*. Washington Referendum Data 2010-2022.

*Notes*. Coefficients given for TAX_INCID and DIST_MILES are estimated using the covariate specification in Table 4, Model 3, without fixed effects for general election timing or year, and no clustering since the model is specified with only one library at a time and weighted by total votes cast in precinct. Weighted WTP shows total votes weighted mean and standard deviation as calculated for each precinct and then averaged for the library. WTP is measured on the means. Distance cost was calculated when the estimated results behaved as expected on tax incidence, even when the statistical significance was null. Stars indicate p values: * = p≤0.05, **=p≤0.01, ***=p≤0.001, +:p=0.053
